# The role of the microbiota in hematological malignancies: A narrative review of mechanisms and therapeutic potential

**DOI:** 10.1016/j.nmni.2026.101805

**Published:** 2026-06-30

**Authors:** Atefeh Valaei, Neda Zahmatkesh, Romina Aghaei, Mahtab Maleki, Maryam Meskini, Seyed Davar Siadat

**Affiliations:** a Department of Molecular Medicine, Pasteur Institute of Iran, Tehran, Iran; b Department of Linguistics, Tarbiat Modares University, Tehran, Iran; c Department of Genetics, Islamic Azad University, Zanjan, Iran; d Department of Cell and Molecular Science, Kharazmi University, Karaj, Iran; e Department of Biology, SR.c, Islamic Azad University, Tehran, Iran; f Department of Mycobacteriology and Pulmonary Research, Pasteur Institute of Iran, Tehran, Iran; g Microbiology Research Center (MRC), Pasteur Institute of Iran, Tehran, Iran; h Student Research Committee, Pasteur Institute of Iran, Tehran, Iran

**Keywords:** Microbiota, Hematological malignancies, Dysbiosis, Immune modulation, Short-chain fatty acids (SCFAs), Fecal microbiota transplantation (FMT), Leukemia, Lymphoma, Multiple myeloma, Graft-versus-host disease (GVHD), Probiotics, Immunotherapy

## Abstract

The human microbiota, particularly the gut microbiome, plays a central role in maintaining immune homeostasis, regulating hematopoiesis, and modulating host metabolism through bioactive metabolites such as short-chain fatty acids (SCFAs), bile acids, and tryptophan-derived compounds. Disruption of this microbial ecosystem (dysbiosis) has emerged as a key contributor to the development and progression of hematological malignancies (HMs), including acute and chronic leukemias, lymphomas, and multiple myeloma. This narrative review synthesizes recent evidence (2022–2025) on the complex bidirectional interactions between the microbiota and HMs, highlighting their biological and clinical significance.

Current evidence indicates that the microbiota influences hematological malignancies through multiple interconnected mechanisms, including immune regulation, inflammatory signaling, maintenance of hematopoietic homeostasis, and microbial metabolite-mediated modulation of the tumor microenvironment. Dysbiosis has been associated with disease progression, increased susceptibility to infections, impaired treatment tolerance, and inferior clinical outcomes. Conversely, chemotherapy, broad-spectrum antibiotics, and hematopoietic stem cell transplantation profoundly reshape microbial communities, further exacerbating dysbiosis and contributing to complications such as graft-versus-host disease following allogeneic transplantation.

Emerging microbiota-targeted interventions, including dietary modulation, probiotics, prebiotics, and fecal microbiota transplantation, show promise for restoring microbial homeostasis and improving therapeutic outcomes. Furthermore, microbiome-derived biomarkers are increasingly being investigated for predicting treatment response, relapse risk, and immunotherapy efficacy. Despite these advances, important challenges remain, particularly in establishing causal relationships, standardizing microbiome profiling, and validating clinical applications through well-designed prospective and randomized studies.

Overall, the accumulating evidence supports the microbiota as a critical determinant of hematological cancer biology and treatment response. Integrating microbiome-based diagnostics and therapeutic strategies into precision hematology may offer new opportunities to improve patient management and long-term clinical outcomes.

## 1 Introduction

### 1.1 Overview of the microbiota and its emerging role in health and disease

**Fig. 1:**
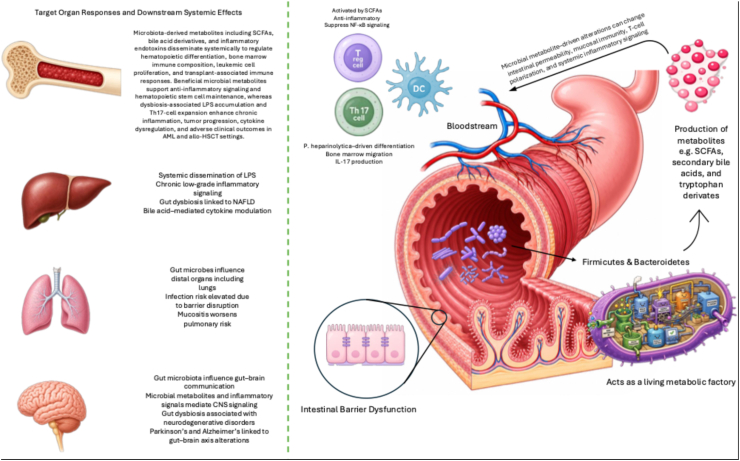
**Gut microbiota dysbiosis and systemic immune regulation in hematologic malignancies and transplantation settings.** The gut microbiota, predominantly composed of *Firmicutes* and *Bacteroidetes*, functions as a dynamic metabolic factory, producing bioactive metabolites, including short-chain fatty acids (SCFAs), secondary bile acids, and tryptophan derivatives. These microbial products enter the bloodstream and modulate intestinal barrier integrity, mucosal immunity, and systemic inflammatory signaling. Beneficial metabolites, such as SCFAs, promote anti-inflammatory responses by suppressing nuclear factor kappa B (NF-κB) signaling and support regulatory T-cell (Treg) activity, whereas dysbiosis-associated microbial alterations promote intestinal permeability, systemic dissemination of lipopolysaccharide (LPS), and chronic low-grade inflammation. Dysbiotic conditions further enhance Th17-cell differentiation and IL-17 production, partly mediated by *Prevotella heparinolytica*, contributing to inflammatory immune polarization. Systemically, microbiota-derived metabolites influence hematopoietic differentiation, bone marrow immune composition, leukemic cell proliferation, and transplant-associated immune responses in acute myeloid leukemia (AML) and allogeneic hematopoietic stem cell transplantation (allo-HSCT). In distal organs, gut dysbiosis is associated with liver inflammation and nonalcoholic fatty liver disease (NAFLD) through bile acid-mediated cytokine modulation, increased risk of pulmonary infections due to barrier disruption and mucositis, and alterations in the gut-brain axis linked to neuroinflammatory and neurodegenerative disorders, including Parkinson's and Alzheimer's diseases.

The term “Microbiome” refers to the collective genomes and metabolic products that interact with host physiology throughout life [[1], [2], [3]]. The intestinal microbiota is mainly composed of *Firmicutes*, *Bacteroidetes*, *Actinobacteria*, and *Proteobacteria* [2,4]*. Firmicutes* and *Bacteroidetes* account for approximately 90% of the intestinal microbiota. These microorganisms play vital roles in digestion, metabolism, immune regulation, and the maintenance of the intestinal barrier (Fig. 1).

The intestine contains about 10^14^ microorganisms, which collectively harbor a genetic content larger than the human genome. There is growing evidence of a bidirectional interaction between human and microbial genes. A healthy intestinal microbiota is generally diverse and functionally constant. However, due to differences in individual physiological states, defining a healthy microbiota is complex [5,6]. The gut microbiota plays a key role in the gut-immune-hematopoiesis axis, producing metabolites such as short-chain fatty acids (SCFAs), bile acids, and tryptophan derivatives, while also maintaining the mucosal barrier by regulating both innate and adaptive immune responses [7,8].

Changes in microbial metabolites and microbial products (such as lipopolysaccharides [LPSs] and peptidoglycan [PG]) can disrupt T-cell polarization, mucosal biology, and tight junctions, and intensify inflammatory pathways while compromising microbial diversity due to drugs, nutrition, or disease [2]. The intestinal microbiota plays an important role in the onset and progression of numerous diseases. Recent research increasingly highlights the crucial role of gut microorganisms in maintaining overall host health. Pathological mechanisms underlying various complex human diseases, such as metabolic, neurological, immune-inflammatory disorders, and cancers, often involve disturbances in the gut microbiota [9]. For instance, an imbalance in intestinal microorganisms (intestinal dysbiosis) can trigger inflammatory and metabolic abnormalities, which are closely associated with obesity, diabetes, inflammatory bowel disease (IBD), and several neurological conditions. The gut microbiome contributes to disease progression both directly and indirectly by regulating host metabolism and immune responses. Consequently, modifying the composition of gut microbes and their metabolic outputs represents a promising approach for diagnosing and treating a wide range of human diseases [5].

Cell death plays a crucial role in preserving tissue homeostasis during normal development and in generating effective responses to pathogens. Recent studies have identified apoptosis, pyroptosis, and ferroptosis as the most recognized forms of programmed cell death. The interaction between the gut microbiota and cell death is highly complex. Cell death can influence microbial invasion by regulating the host immune response, maintaining the intestinal barrier, and providing protection against pathogenic invasion. A balanced gut microbiota helps to suppress excessive inflammation, reduce unnecessary cell death, and preserve overall tissue integrity. However, when the normal regulation of the gut microbiota is disrupted, the excessive proliferation of harmful microorganisms can trigger abnormal cell death, which, in turn, promotes inflammation and contributes to the progression of related diseases. The gut microbiome plays a crucial role in the onset and development of inflammatory disorders and cancers by affecting cell death pathways, such as pyroptosis and ferroptosis, through its metabolic products [5].

Nevertheless, only a limited number of studies have explicitly focused on the mechanisms linking intestinal microbiota with necrosis-related diseases. Most existing research emphasizes intestinal conditions like colorectal cancer (CRC) and IBD. Although some studies have explored possible connections between gut microbes and other organs, their conclusions remain inconclusive. This uncertainty may be explained by the complex and dynamic nature of the gut-organ axis, which encompasses multifaceted interactions between the intestine and other organs. Moreover, metabolites produced by gut microbes can influence distant organs such as the brain, liver, and lungs [5] (Fig. 1).

The human body hosts a complex ecosystem of numerous microorganisms that live both inside and on its surface, collectively referred to as the human microbiota. While the majority of these microorganisms inhabit the gastrointestinal tract, others are also found on the skin, in the lungs, vagina, and mammary glands. The gastrointestinal tract, with an estimated surface area of about thirty-two square meters, represents one of the largest sites of interaction between the host, antigens, and environmental agents. Various factors, including physiological traits, chemical and nutritional gradients, and the well-organized host immune response, shape the diversity of microbial communities within the mammalian gut. The intestinal microbiota and the human host maintain a bidirectional relationship in which the immune system plays a key role in sustaining homeostasis with microbial populations. This mutual interaction allows symbiotic microorganisms to influence mammalian immune function. Alterations in the normal gut microbiota, known as dysbiosis, have been identified as a major contributor to the development of numerous diseases. Recent studies have reported that nearly 13% of cancer cases worldwide are associated with microorganisms. Furthermore, the human microbiota can affect both the efficacy and toxicity of cancer treatments, including chemotherapy, radiotherapy, and, more recently, immunotherapy [10].

It is important to note that the majority of evidence linking gut microbiota composition to HMs derives from observational, cross-sectional, and preclinical studies. As such, most reported associations between dysbiosis and disease outcomes are associative rather than causal. Reverse causation, whereby the malignancy or its treatment causes the observed microbial dysbiosis rather than the reverse, cannot be excluded in these study designs. Throughout this review, causal language is reserved for findings supported by randomized or mechanistically validated preclinical evidence; associative language is used for findings supported by observational evidence.

### 1.2 Introduction to HMs and their clinical significance

**Fig. 2:**
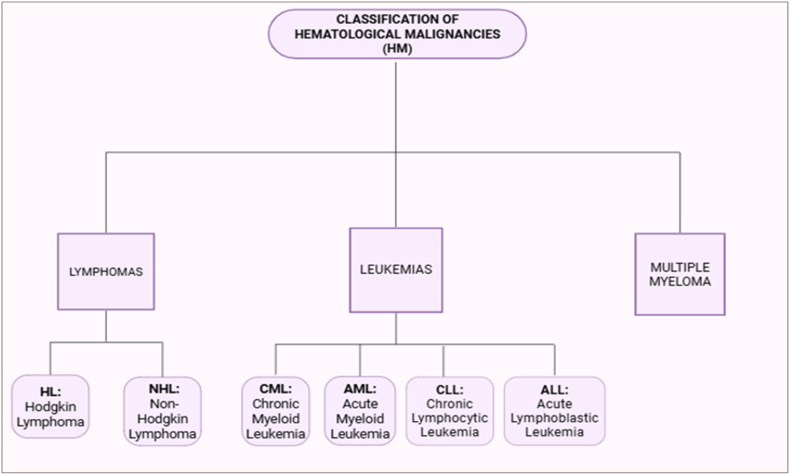
Tree diagram—high-level classification of hematologic malignancies.

There is a diverse group of Neoplasms originating from lymphoid and myeloid lineages, including Leukemias, Lymphomas, and multiple myeloma (MM) (Fig. 2).

**Fig. 3:**
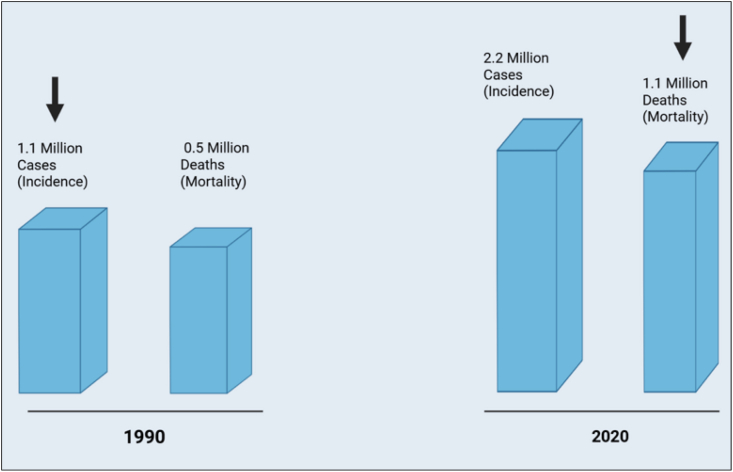
Global trend of incidence and mortality inHMs (1990 vs. 2020).

The World Health Organization (WHO) classification (fifth edition), reflecting rapid advances in molecular research, integrates histology with cytogenetic and molecular markers to refine diagnostic criteria [[11], [12], [13]]. Has represented a significant global public health burden. At the same time, mortality trends vary among different subgroups. Innovative drugs and supportive care have improved survival in many settings. A recent worldwide analysis shows that the incidence of HMs has steadily increased since 1990 due to demographic changes and changing environmental exposure [11,12] (Fig. 3).

Population-based registers in high-income countries, despite an increasing incidence rate, show a decrease in mortality for several HM categories, reflecting the effect of accurate diagnosis, immune modulators, and proteasome inhibitors in myeloma, Bruton's tyrosine kinase and BCL2 inhibitors in B-cell malignancies, and improvement in transplant and prevention conditions of GVHD [11]. However, there are significant differences across regions and health systems, and treatment-related complications, especially in allo-HSCT, remain significant.

#### 1.2.1 Gut microbiota inHMs

Hematopoiesis regulates the generation of immune cells by guiding hematopoietic stem cells (HSCs) to differentiate into multiple progenitor lineages. A range of intrinsic and extrinsic factors influences this process. Among external regulators, the microbiota, diverse microorganisms residing throughout the body, play a significant role. By releasing structural components and bioactive molecules, the microbiota profoundly influences host homeostasis and supports the normal functioning of various organs [14].

Alterations in the gut microbiota have been investigated in both mouse models and clinical settings among adult and pediatric patients with hematologic disorders. Evidence suggests that survivors of Hodgkin lymphoma (HL) have a reduced abundance of rare gut microorganisms compared with their healthy twin counterparts. It has been observed that the gut microbiota can induce oxidative stress, which may affect carcinogenesis and influence several pathways involved in lymphogenesis. Symbiotic bacteria are known to contribute to Th17 cell differentiation, which plays a crucial role in inflammatory processes by producing IL-17. In particular, the commensal bacterium *P. heparinolytica* has been shown to promote the differentiation of gut-resident Th17 cells. These cells can migrate to the bone marrow, where they participate in tumor progression. A human study examining the relationship between gut flora alterations and disease relapse following allo-HSCT found that *Eubacterium* was associated with a reduced risk of multiple myeloid relapses after transplantation. Furthermore, several studies have documented a marked reduction in bacterial diversity among patients with acute lymphoblastic leukemia (ALL) and acute myeloid leukemia (AML) [10].

Several studies have linked anaerobic-active regimens to worse outcomes and a higher risk of HGVD, suggesting that antibiotic exposure is an important and potentially modifiable factor in these ecological harms. Given these findings, the field is now evaluating restorative interventions, including fecal microbiota transplantation (FMT), which maintains the microbiome in transplantation groups [15].

### 1.3 Objectives of the narrative review and study design

Given the growing importance of this field, this review article aims to provide a comprehensive overview of the mechanisms by which the microbiota influences the initiation, progression, and treatment of HMs.

In this regard, we first review the pathophysiology of central HMs, then investigate the role of the microbiome in modulating the immune system and metabolism, and finally discuss the therapeutic potential of microbiota-based interventions, including probiotics and FMT. Currently, structured monitoring of gut diversity and dominance as emerging biomarkers in transplant programs, nutritional strategies to support SCFA-producing species during neutropenia, and monitoring to avoid unnecessary anaerobic antibiotics when clinically safe are needed. However, we caution that widespread use of microbiome therapies in HMs requires strong evidence, including randomized studies with significant clinical outcomes (e.g., grade III–IV acute GVHD, infection-related mortality), standardized ecological measures (diversity, metabolite readouts), and pharmacologic care for immunocompromised hosts [15].

Platform-style double-anonymized FMT trials and microbiota-free antibiotic regimens currently being conducted during transplantation points the way forward in this field [16,17].

Finally, we place the HM-microbiome relationship within the broader framework of immunology and cancer. Although HM care provides a particularly elucidated natural experiment due to significant pathogenetic disturbances and distinct immunological endpoints, many ecological and metabolic principles apply to oncology. Using these features with careful trial design, the microbiome could be beneficial not only to patients undergoing allo-HSCT but also to those receiving CAR-T cell agents or maintenance therapies, where microbial ecology is associated with the risk of infection, mucosal injury, and metabolic toxicities.

This work is structured as a narrative review, a study design appropriate for synthesizing a broad and rapidly evolving body of evidence spanning multiple disciplinary fields, including microbiology, immunology, hematological oncology, and clinical transplantation medicine. Narrative reviews are particularly suited to emerging interdisciplinary areas where the evidence base is heterogeneous in design, scale, and methodology, and where a formal systematic review or meta-analysis would be constrained by inconsistency in outcome reporting and the absence of standardized microbiome profiling methods across studies. Consistent with the guidelines of the *International Committee of Medical Journal Editors* (ICMJE) and the *SANRA* checklist for narrative reviews [18], this review was planned with explicit literature search parameters, defined scope boundaries, and a structured approach to evidence appraisal. The search period was set from January 2018 to March 2025 to capture literature published since the emergence of high-throughput microbiome sequencing as a standard research tool. Seminal foundational studies published before 2018 were retained where they represent primary evidence not superseded by more recent work (e.g., Taur et al., 2014 [19] on microbiome diversity and allo-HCT mortality; Jenq et al., 2015 [20] on intestinal *Blautia* and GVHD). Reference lists of included articles were manually screened to identify additional relevant publications not captured in the primary database search. Search queries were constructed using Medical Subject Headings (MeSH) terms in PubMed and equivalent controlled vocabulary in Scopus and Web of Science, combined with free-text terms. Boolean operators (AND/OR) were applied to combine domain-specific term clusters. Searches were limited to peer-reviewed publications in English. No restrictions were applied to country of origin, study population size, or study setting at the initial search stage.

## 2 Overview of HMs

According to reporting of global cancer statistics, the incidence of ALL, AML, Chronic Lymphocytic Leukemia (CLL), and Chronic Myeloid Leukemia (CML) is approximately 1.7–4.6, 1.2–4.2, 2.0–5.0, and 0.8–2.1 individuals per 100,000 population, respectively, and continues an upward trend in recent years [21,22].

Globally, the incidence of HMs has increased from 1990 to 2019. The age-standardized incidence rate also showed an upward trend. Our analysis of global disease data shows a continuous and statistically significant increase in the incidence of HMs over the past three decades. This upward trend highlights a growing public health challenge worldwide [11].

Therefore, HMs include a diverse group of cancers, like leukemia, lymphoma, and MM, that are important in global public health due to their increasing prevalence and clinical complexities. These diseases originated from HSCs, not only profoundly affecting the body's immune systems, but also the standard treatment, such as chemotherapy and stem cell transplantation, severely disrupts the balance of the body's microbial ecosystem. Therefore, understanding the interaction between the microbiota and the pathophysiology of these cancers can open new horizons for diagnosis, prognosis, and the design of novel treatment interventions [11].

Genetic and environmental factors influence the development of HMs. Studies in pediatric populations have identified several genetic syndromes with a lower risk of ALL. These include Down syndrome, Fanconi anemia, Bloom syndrome, ataxia telangiectasia, and Nijmegen breakage syndrome [23]. Other risk factors for ALL include exposure to ionizing radiation, pesticides, specific chemical solvents, and viral infections such as Epstein–Barr virus and human immunodeficiency virus [24]. HMs have arisen from defective HSCs and are characterized by a wide range of somatic mutations and chromosomal abnormalities [25]. Clonal hematopoiesis (CH) refers to the emergence of a genetically distinct population of blood cells resulting from the clonal expansion of mutated HSCs, occurring in the absence of any hematologic disease or clinical manifestations [26,27]. CH has been linked to an elevated risk of developing leukemia, although it does not typically progress to hematologic malignancies [28].

### 2.1 Leukemia: classification and pathophysiology of major subtypes (ALL, AML, CLL, CML)

This form of blood cancer usually originates in the bone marrow (BM), where it disrupts normal blood cell production and primarily affects white blood cells. Leukemia can manifest as either acute, with rapid progression, or chronic, with a slower course [29].

#### 2.1.1 Acute myeloid leukemia (AML)

AML is a type of hematological malignancy in which abnormal myeloid cells (a type of white blood cell) rapidly multiply in BM [29]. In AML, human and animal evidence have reported a causal relationship between dysbiosis and disease progression. Induction of dysbiosis with antibiotics promotes AML progression in mouse models, and FMT reverses this process. In addition, in AML patients undergoing chemotherapy, severe disruption of microbiota composition or function and deficiency of the intestinal barrier have been reported, which correlate with infectious and inflammatory complications [7,30,31]. Interleukin-6 (IL-6) and tumor necrosis factor-alpha (TNF-α) were elevated in the serum of AML patients [32].

#### 2.1.2 Acute lymphoblastic leukemia (ALL)

ALL is characterized by the malignant transformation and proliferation of lymphoid progenitor cells within the BM, blood, and extramedullary tissues [33]. Chromosomal and genetic abnormalities contribute to the differentiation and proliferation of lymphoid progenitor cells [34]. Elevated serum levels of IL-6 and TNF-α were found in ALL patients [32,35]. In ALL, especially in children, the diversity and composition of the microbiota before treatment initiation are correlated with infectious outcomes and chemotherapy tolerance. Specific patterns of *Proteobacteria*/*Enterococcus* predominance and decreased SCFA producers predict infection and chemotherapy risks. Also, although gut composition can return to a healthy state after chemotherapy ends, changes can persist [36,37]. Another study examined significant differences in the bacterial composition of fecal samples from ALL patients compared to healthy controls. Using 16S rRNA quantitative arrays and bioinformatic analyses, the researchers identified a notably higher abundance of *Bacteroides clarus* in the fecal specimens of ALL patients [38].

#### 2.1.3 Chronic lymphoblastic leukemia (CLL)

This type of leukemia progresses slowly and is most common in adults. The elevated TNF-α and IL-6 were observed in CLL patients [39]. In chronic lymphocytic leukemia (CLL), human data and animal models indicate that the composition and diversity of the microbiota are associated with the onset/course of the disease. A group of patients with lower diversity has distinct bacterial patterns that correlate with immune state and disease parameters. These findings reinforce the influence of the microbiota on the B-cell tumor microenvironment [40].

B-cell receptor inhibitors, used as targeted therapies, have been shown to increase Bacteroidia abundance in patients with chronic CLL [41]. This finding further underscores the link between gut microbiota and leukemia.

#### 2.1.4 Chronic myeloid Leukemia (CML)

Chronic myeloid leukemia (CML), a more slowly progressing form of myeloid leukemia, affected approximately 34,200 individuals worldwide in 2017. Its global incidence is estimated at around 0.8–2.1 cases per 100,000 population, and HMs has shown an upward trend in recent years [21,22]. CML develops as a result of the Philadelphia chromosome abnormality, which is caused by the BCR-ABL1 fusion gene generated through the chromosomal translocation t ([9,22]) (q34; q11.2) [42]. The BCR-ABL1 fusion gene promotes uncontrolled cellular proliferation by constitutively activating the BCR-ABL1 tyrosine kinase [43]. The levels of IL-6, transforming growth factor-alpha (TGF-α), and TNF were increased in the serum of CML patients [44]. In CML, standard treatment includes targeted tyrosine kinase inhibitors. One of the commonly used inhibitors is imatinib, and more recently, asciminib [45,46]. Historically, interferon alpha (INF-α) was used to treat CML, but its clinical use was limited by significant adverse effects [46]. Furthermore, CML patients may also receive allogeneic stem cell transplantation as a therapeutic option. Beyond leukemia-specific therapies, some treatments have been shown to influence the gut microbiota. For instance, antibiotic administration that reduces intestinal microbial populations can enhance the anti-leukemic activity of APO866. Decrease of Nicotinamide-converting bacteria (e.g., *Escherichia coli*) to prevent nicotinamide adenine dinucleotide (NAD) rescue and restore APO866 efficacy. As a result, APO866 (a NAMPT inhibitor) restores sensitivity to chemotherapy drugs [47]. The relationship between the BCR-ABL1 oncogenic pathway and gut microbiota in CML is uniquely bidirectional and mechanistically specific in ways not yet described for other HMs. The BCR-ABL1 fusion protein, generated by the t (9;22) (q34; q11.2) translocation, drives constitutive tyrosine kinase activation and downstream engagement of STAT3, MAPK, and PI3K/AKT signaling cascades, leading to uncontrolled myeloid proliferation and resistance to apoptosis. These same signaling pathways regulate intestinal epithelial homeostasis and mucosal immune function; thus, BCR-ABL1 activity may contribute to gut barrier dysregulation independently of chemotherapy exposure [47].

More directly, a critical pharmacological intersection has been established between gut microbiota and the efficacy of NAMPT inhibitors, a class of agents targeting NAD biosynthesis in CML cells, as reported by Ref. [47]. Nicotinamide phosphoribosyl transferase (NAMPT) is the rate-limiting enzyme in NAD + biosynthesis from nicotinamide (NAM), and its inhibition (e.g., by APO866) induces leukemia cell death by NAD depletion. However, gut bacteria, including *E.* *coli* and other NAM-converting species, possess enzymatic machinery to convert NAM into nicotinic acid (NA), which fuels an alternative NAD-synthesizing pathway that bypasses NAMPT inhibition entirely. In murine leukemia models, antibiotic-mediated depletion of these NAM-converting bacteria restored APO866 anti-leukemic efficacy, establishing a direct causal link between microbiota composition and therapeutic resistance in CML. This finding implies that the clinical failure of NAMPT inhibitors in early trials may have been partly attributable to microbiota-mediated NAD rescue, a modifiable variable that could be targeted through antibiotic co-administration or dietary NAM restriction.

Beyond treatment metabolism, TKI therapy itself has gastrointestinal consequences that reciprocally alter microbiota composition. Imatinib, nilotinib, dasatinib, and other TKIs frequently cause diarrhea, nausea, and altered intestinal motility, creating conditions for secondary dysbiosis. An ongoing prospective clinical trial (NCT06724536) is evaluating whether specific pre-treatment microbiome signatures are associated with the probability of achieving deep molecular response to TKIs in CML patients, a line of investigation that may enable microbiome-based prediction of treatment outcomes in this molecularly defined disease.

Conventional observational studies have identified links between alterations in the gut microbiota and the development of leukemia. In a prospective clinical study involving 29 patients with AML, 17 with CML, and 33 healthy controls, researchers observed that the phyla *Actinobacteria*, *Acidobacteria*, and *Chloroflexi* were more abundant in patients with AML and CML than in healthy controls. In contrast, *Tenericutes* were notably decreased in CML patients [48]. Pötgens et al. (2024) observed a relative decrease in the genus *Eubacterium*, particularly *Eubacterium eligens*, in AML patients, with levels reduced by up to threefold [49].

### 2.2 Lymphoma: characteristics of hodgkin and non-hodgkin lymphomas

This type of leukemia affects the lymphatic system and can begin in a lymph node. Lymphoma is classified as non-Hodgkin lymphoma (NHL) or HL [50].

#### 2.2.1 Hodgkin lymphoma (HL)

It is characterized by the presence of abnormal cells called Reed-Sternberg (HRS) cells in a biopsy sample, which are derived from germinal center B cells but have lost the typical B-cell phenotype [51]. According to molecular studies, one of the common causes of HL pathogenesis is activation of the nuclear factor kappa B (NF-κB) pathway (for example, through amplification of REL or TNFAIP3 mutations) [52]. *F. prausnitzii* was removed from the intestinal microbiota community in HL patients [53]. Also, increased serum levels of IL-6 and TNF-α were found in HL patients [54,55]. Classical HL is distinguished from nodal lymphocyte-predominant HL by differences in clinical presentation, morphology, immunophenotype, and genetic features [51].

#### 2.2.2 Non-Hodgkin lymphoma (NHL)

Several types of this lymphoma arise in different cells of the lymphatic system [51]. NHL represents a heterogeneous group of lymphoid malignancies arising from B or T lymphocytes and includes B-cell subtypes (for example, diffuse large B-cell lymphoma (DLBCL), follicular lymphoma (FL), and mantle cell lymphoma [MCL]) and T-cell subtypes [52], that have diverse clinical behaviors ranging from slow growing to highly aggressive. DLBCL is the most common of NHL subtypes [56]. Increased *Escherichia*, *Shigella*, and *Lachnospira*, and reduced *F*. *prausnitzii* have been observed in NHL patients [53]. Also, Elevated serum levels of IL-6 and IL-10 were significantly seen in DLBCL patients [57]. The WHO classification system classifies lymphoma based on its morphology, immunophenotype, genetic features, and clinical presentation [51]. Distinct molecular changes, such as chromosomal translocations, gene mutations, and pathway disruptions, indicate different NHL subtypes and affect prognosis and therapeutic approaches. Clinically, HL often manifests as painless lymphadenopathy accompanied by systemic B symptoms, including fever, nocturnal sweating, and unintentional weight loss, whereas the presentation of NHL varies by subtype. Treatment strategies differ significantly between HL and NHL, reflecting their unique biology and clinical courses [51]. Depending on the stage of disease and toxicity of treatment, HL, especially classical lymphoma, is highly treatable with modern combined therapy [58].

### 2.3 MM: pathogenesis and clinical features

MM is a clonal malignancy of plasma cells, characterized by the accumulation of abnormal plasma cells in the BM, the production of monoclonal immunoglobulin, and clinical manifestations such as bone lesions, bone pain, lytic lesions, anemia, renal impairment, hypercalcemia, and increased susceptibility to infections [59]. Clinically, patients are often observed with anemia, renal impairment, and susceptibility to infections [42]. The disease involves various genetic abnormalities, including chromosomal translocations affecting the immunoglobulin heavy chain (IGH) locus, mutations in oncogenes and tumor suppressor genes, and dysregulation of key signaling pathways such as NF-κB, MAPK, and PI3K/AKT [59]. Interactions between myeloma cells and the BM microenvironment, including stromal cells and extracellular matrix proteins, further contribute to disease progression. In other words, interactions with the BM niche, through cytokines such as VEGF, IL-6, and NF-κB, and through the cytokine-driven microenvironment and adhesion molecules, promote bone remodeling, resistance, and survival, and mediate the course of MM [59]. Clinically, MM appears with symptoms associated with BM infiltration, such as bone pain and fractures, fatigue associated with anemia, renal dysfunction, and immunosuppression leading to infection. Advances in understanding MM biology have led to new therapeutic agents that target both myeloma cells and their supportive microenvironment, thereby improving patient outcomes [59]. MM is a malignancy of terminally differentiated plasma cells that accumulate in the BM, causing widespread bone disease, anemia, renal dysfunction, and immunodeficiency. The genetic landscape of MM is complex, with early events such as IGH locus translocations and secondary genetic mutations driving disease progression. The BM microenvironment, including mesenchymal stromal cells, osteoclasts, osteoblasts, endothelial cells, and immune cells, supports myeloma cell growth and is resistant to therapy. Clinically, patients present with CRAB features (hypercalcemia, renal failure, anemia, bone lesions) that reflect the field biology of the disease. Therapeutic strategies that target both malignant plasma cells and their microenvironment are part of current therapeutic approaches [60]. The median age at diagnosis is approximately 70 years. Advances in therapy, including proteasome inhibitors, immunomodulatory agents (IMiDs), monoclonal antibodies, and autologous stem cell transplantation, have significantly improved patient survival [42].

Table 1 below provides a systematic cross-comparison of the key microbial signatures, major depleted and enriched taxa, dominant altered metabolites, and principal immune/inflammatory pathways reported for each hematological malignancy subtype covered in this review.

**Table 1:** Cross-comparison of gut microbiota signatures, metabolite alterations, and immune pathway disruptions across major HMs.

Disease	Key Depleted Taxa	Key Enriched Taxa	Dominant Altered Metabolites	Principal Immune/Inflammatory Pathways	Tumor-Specific Driver Link	References
**AML**	*Eubacterium eligens* (up to 3-fold reduction)^a^; *Faecalibacterium prausnitzii*^a^; *Lachnospiraceae* NC2004 group; *Prevotella 9*; *Megamonas*	*Streptococcus* spp.^a^; *Actinobacteria*, *Acidobacteria*, *Chloroflexi* (phylum level)	Reduced butyrate and SCFAs^a^; elevated LPS (barrier disruption)^a^; altered amino acid and lipid metabolome; elevated formic acid (potential biomarker)	TLR4-NF-kB activation (LPS-driven)^a^; impaired Treg/Th17 balance; elevated IL-6 and TNF-alpha^a^; myeloid-derived suppressor cell expansion; compromised innate immune surveillance	Dysbiosis-driven LPS translocation exacerbates myeloid inflammatory signaling; HDAC inhibition by butyrate depletion may reduce tumor suppressor gene expression.	[7,48,49,61]
**ALL**	SCFA-producing *Firmicutes*^a^; overall alpha-diversity significantly reduced^a^; *Bifidobacterium* spp.	*Proteobacteria* (phylum)^a^; *Enterococcus* spp.^a^; *Bacteroides clarus* (markedly elevated vs. healthy controls)	Reduced SCFAs (especially butyrate)^a^; altered sphingolipid and lipid metabolome; tryptophan-kynurenine pathway dysregulation	IL-6 and TNF-alpha elevation^a^; Th17 expansion; impaired mucosal IgA; reduced CD8^+^ cytotoxic T-cell activity; increased infection and chemotherapy-related complication risk	Pre-treatment Proteobacteria/Enterococcus predominance predicts infectious outcomes and chemotherapy tolerance; barrier disruption correlates with febrile neutropenia.	[[36], [37], [38],^62^]
**CML**	*Tenericutes* (phylum)^a^; nicotinamide-converting bacteria (e.g., *E. coli*) relevant to the NAD pathway	*Actinobacteria*, *Acidobacteria*, *Chloroflexi* (phylum)^a^; variable genus-level changes	Altered nicotinamide (NAM)/nicotinic acid (NA) balance via bacterial NAM-to-NA conversion, undermines NAMPT inhibitor (APO866) efficacy; elevated IL-6, TGF-alpha, TNF.	IL-6/STAT3 activation^a^; TGF-alpha-mediated immune suppression; BCR-ABL1-driven myeloid expansion creating pro-inflammatory cytokine milieu	BCR-ABL1 tyrosine kinase constitutively activates downstream STAT3, MAPK, and PI3K/AKT; gut bacteria rescue NAD synthesis (via NA), directly blunting NAMPT inhibitor antileukemic activity. TKI gastrointestinal side effects bidirectionally alter microbiota.	[47,48]
**CLL**	Overall alpha-diversity reduced; *Faecalibacterium prausnitzii*^a^; key SCFA producers.	Bacteroidia increased (notably following BCR inhibitor therapy); distinct genus-level patterns correlate with disease stage.	Reduced butyrate^a^; altered bile acids; dysregulated B-cell activating factor (BAFF)-related signaling metabolites	TNF-alpha and IL-6 elevation^a^; BCR signaling-TLR crosstalk; NF-kB activation; skewed B-cell tumor microenvironment; impaired anti-tumor T-cell surveillance	BCR inhibitors (ibrutinib/BTK inhibitors) alter gut microbiota composition (Bacteroidia increase); TLR ligands from gut microbiota may co-stimulate BCR signaling in malignant B cells, potentially sustaining tumor proliferation	[[39], [40], [41]]
**Hodgkin Lymphoma (HL)**	*F. prausnitzii*^a^ (removed from community); rare gut taxa in long-term survivors (vs. healthy twins)	Inflammatory taxa; limited human-specific data; oxidative stress-associated microbial shifts	Reduced anti-inflammatory SCFAs; microbiota-induced oxidative stress metabolites linked to lymphomagenesis	NF-kB pathway activation (REL amplification/TNFAIP3 mutations)^a^; IL-6 and TNF-alpha elevation^a^; microbiota-driven oxidative DNA damage contributing to Reed-Sternberg cell survival	NF-kB is constitutively active in HRS cells; gut-derived LPS and inflammatory taxa may sustain NF-kB activation via TLR4; microbiota-induced Th17 differentiation may contribute to inflammatory TME.	[10,53,54]
**Non-Hodgkin Lymphoma (NHL/DLBCL)**	*F. prausnitzii*^a^; butyrate-producing *Firmicutes*	*E. coli* and *Shigella*^a^; increased *Lachnospira*^a^; elevated IL-6 and IL-10 (DLBCL)	Reduced SCFAs^a^; elevated pro-inflammatory cytokine metabolites (IL-6, IL-10 in DLBCL); microbiota-driven modulation of kynurenine pathway	IL-6 and IL-10 elevation (DLBCL)^a^; BCL2 dysregulation; NF-kB pathway activation; impaired anti-tumor innate immune surveillance; microbiota-induced Th17 cell expansion	Chromosomal translocations (e.g., BCL2, MYC) define NHL subtypes; gut-derived inflammatory cytokines (IL-6) may sustain JAK-STAT and NF-kB signaling in lymphoma cells; Prevotella-driven Th17 pathway implicated across B-cell lymphomas	[10,53,57]
**Multiple Myeloma (MM)**	SCFA-producing taxa (*F. prausnitzii*^a^, *Bifidobacterium* spp.); *Eubacterium* spp. (post-HSCT protective); *Prevotella melaninogenica* (restrains MM progression)	*Prevotella heparinolytica*^a^ (drives Th17 migration to BM); nitrogen-recycling bacteria (*Klebsiella pneumoniae*, promotes glutamine synthesis); pathogenic species promoting chronic antigenic stimulation	Reduced SCFAs^a^; increased glutamine/ammonia from nitrogen-recycling microbiota; altered bile acids; VEGF, IL-6, and IL-17 as downstream inflammatory metabolite mediators	IL-6/NF-kB axis^a^; IL-17 (Th17 migration from gut to BM)^a^; VEGF-driven angiogenesis; eosinophil activation; PI3K/AKT and MAPK pathway dysregulation; bone marrow microenvironment remodeling	*P. heparinolytica* drives gut Th17 differentiation; these cells migrate to the BM, where IL-17 promotes plasma cell proliferation (Vk^a^MYC model). Dysbiosis-driven IL-6/NF-kB activation directly sustains myeloma cell survival and proteasome inhibitor resistance	[10,63,64]

a Feature observed across three or more hematological malignancy subtypes (shared signature). AML, acute myeloid leukemia; ALL, acute lymphoblastic leukemia; CML, chronic myeloid leukemia; CLL, chronic lymphocytic leukemia; HL, Hodgkin lymphoma; NHL, non-Hodgkin lymphoma; DLBCL, diffuse large B-cell lymphoma; MM, multiple myeloma; SCFA, short-chain fatty acid; TME, tumor microenvironment; BM, bone marrow; LPS, lipopolysaccharide; TLR, Toll-like receptor; NAMPT, nicotinamide phosphoribosyltransferase; HDAC, histone deacetylase; BCR, B-cell receptor; BTK, Bruton's tyrosine kinase.

## 3 The human microbiota: composition and functions

### 3.1 Microbial ecosystems: diversity and roles of gut, oral, and other microbial communities

The human microbiome includes a trillion microorganisms residing in various body habitats, such as the intestine, skin, vaginal, oral, lung, and genitourinary systems. These microbial communities exhibit significant diversity and dynamic interactions, which are essential for maintaining host homeostasis [65]. The gut microbiome is the largest microbial ecosystem in the human body. Microorganisms colonize the entire epithelial tissue and maintain functional relationships with host physiology. The human microbiota is primarily composed of bacteria. The microbiome genome is 100-fold that of the Human genome; however, its total number is comparable to that of mammalian cells [66]. In addition to bacteria, the human microbiota includes archaea, viruses, and mycoses, with different ratios at different colonization sites. The gut bacteriome of mammals is mainly composed of 6 branches: *Actinobacteria, Bacteroides, Firmicutes, Verrucomicrobia, Proteobacteria, and Fusobacteria*, with *Bacteroides* and *Firmicutes* accounting for almost 90% of the total communities [67]. These microorganisms, together, perform critical functions such as vitamin production, nutrient metabolism, and immunomodulation, and also defend against colonization by pathogens [68]. Although microbiome composition is relatively stable across the adult lifespan, species and functional diversity of these communities differ among individuals in response to external factors such as health status, diet, geographic location, drug use (especially antibiotics), age, environment, and genetic host [69,70]. The secondary microbial environment of the oral cavity is diverse. It has been shown that there are over 700 types of bacteria that form very structured biofilms on the surfaces of the tongue, teeth, and gums [71]. The oral microbiome also comprises a complex community that plays an important role in maintaining oral and tooth health and affects systemic health [65]. Environmental factors, including nutrition, health, and saliva, strongly influence the composition of oral microbes. Systemic disease, tooth decay, and periodontal disease are closely associated with disorders in this group [72,73].

Recently, studies have highlighted important linkages between oral and gut microbial communities. Oral bacteria swallowed with saliva can be translocated into the intestine, where they can sometimes persist and alter the modulation of intestinal microorganisms. This gut-oral axis has been associated with inflammatory and chronic disorders of the digestive system and shows that oral and tooth health may be important for maintaining systemic microbial modulation [25].

Microbial ecosystems are found in regions beyond the host, including homes, hospitals, and workplaces. Microbial communities in these environments are affected by external factors and human inhabitants [26]. Ecological theories are increasingly used in microbiome studies to better understand how to develop and sustain such ecosystems. Accumulation and tolerance of microbes are shaped by accident mechanisms such as accident colonization, host immune responses, and nutrition. Interventional design can improve or restore microbial modulation for human well-being, a goal achievable through an ecological perspective on microbial ecosystems [27].

### 3.2 Physiological roles: modulation of immunity, metabolism, and inflammation

Microbiota helps through several pathways in development and host immune system function:

1. Growth and maturity of adaptive and innate immune through lymphocystis and macrophage [[74], [75], [76]]. Immune responses are formed and modulated to a large extent by the human microbiome. The immune system is trained by the initial colonization of the intestine and other mucosal surfaces to provide adequate defense against infections and to reinforce tolerance to symbiotic organisms. Immune hemostasis is maintained by symbiotic organisms that control the balance between pro-inflammatory and anti-inflammatory immune cells, including T helper (Th) and regulatory T (Treg) subsets [77,78].
2. Production of active metabolites, SCFA, derivations of tryptophan, and bile acids, effect on host metabolism, mucosal barrier, and inflammatory genes expression, and decrease inflammatory pathways and increase Treg-cells activation by regulating inflammatory responses [[74], [75], [76],^79^].
3. Induce or suppress local inflammatory responses or systemic [[74], [75], [76]]. In addition to immunity, microbiota have a significant impact on immune and host metabolism. Gut microbes control fat and carbohydrate metabolism and help to harvest energy from indigestible polysaccharides [80]. Chemical products produced directly by the microbiome can impact systemic inflammation; for example, in addition to regulating metabolism, SCFAs suppress a critical signaling pathway in inflammation, NF-κB [81]. Metabolic diseases such as obesity, diabetic type 2, and non-alcoholic fatty liver disease have a close relationship with changes in microbial composition [82]. The microbiome can play a role in both disease prevention (for example, resistance to pathogens and metabolism of glucose/lipids) and in shaping the response to treatments (such as immunotherapies and anticancer therapies) [[74], [75], [76]]. Disturbance in gut microbiota composition can impair immune regulation and lead to chronic inflammation, and can play a role in inflammatory gut disease and cancers. Host-microbiota interactions modulate systemic metabolism by affecting energy storage and harvesting [83].

### 3.3 Dysbiosis and disease: implications of microbial imbalances in systemic health

Healthy human microbiota compositions are considered symbiotic. Pathogenic microorganisms, also known as pathogens, are mainly acquired through infectious routes and are therefore usually transient. *Streptococcus pneumoniae* [84], *Staphylococcus aureus* [85], and *Candida albicans* [86] are members of the normal microbiota and can transform into pathogenic forms under specific environmental conditions. The interactions and modulation among community microbial members are critical for health. The genomic and metabolic contributions of the microbiota influence various biological processes, including immunity, metabolism, endocrine function, and nervous system regulation. As a result, the microbiota plays a critical role in maintaining homeostasis. Numerous studies have linked alterations in microbial composition to disease states using genomic and metabolic analyses, leading to the concept of “dysbiosis” [14]. Dysbiosis describes an imbalance in microbial communities that disrupts the normal symbiotic relationship between the host and its microbiota. Immune and metabolic problems have been significantly affected by this imbalance. As dysbiosis weakens the intestinal barrier, microbial components, such as LPS, can enter the bloodstream, leading to low-grade systemic inflammation [87]. In addition to metabolic regulation, Dysbiosis affects immunological response in ways that predispose individuals to autoimmune and inflammatory diseases. Enrichment of pro-inflammatory species promotes the activation of Th17 and other effector cells. Reduced abundance of beneficial bacteria producing fatty acids with short chains (SCFA), such as *F. prausnitzii*, inhibits anti-inflammatory pathways [88].

Rheumatoid arthritis, multiple sclerosis, and IBD are chronic diseases that are exacerbated by these changes in immunological hemostasis [89]. Therefore, microbial dysbiosis plays a role in a wide range of diseases, including autoimmune disorders, metabolic syndrome, and cancers. Also, microbial changes can alter an individual's response to drugs and the toxicity of treatments. Recent studies (2024-2025) have provided a review of the evidence on causality and inflammatory/metabolic pathways that reinforces these relations [75,90,91]. Changes in the microbiota can exacerbate gut inflammation, disrupt the intestinal barrier, and affect distant organs through the gut-organ axis [92].

Factors that contribute to microbial imbalance include lifestyle, drugs, inflammation, stress, and malnutrition, the last of which has been shown to significantly affect the microbial community [93,94]. Restoring microbial balance through prebiotics, probiotics, and FMT is promising for treating dysbiosis-related diseases [92].

#### 3.3.1 Dysbiosis and systemic diseases relationships

Growing evidence shows that the effect of microbial imbalance goes beyond the gut. For example, changes in oral microbiota have a close relationship with periodontal disease, and they may also play a role in allowing infections and inflammatory mediators to spread throughout the body, as inflammatory diseases (including IBD and rheumatoid arthritis) [95], metabolic and cardiovascular disorders (like type 2 diabetes and atherosclerosis) [93,94]. Neurodegenerative diseases such as Parkinson's and Alzheimer's have been associated with a biological imbalance in which inflammatory signals and microbial metabolites interact with the gut-brain axis [96].

**Fig. 4:**
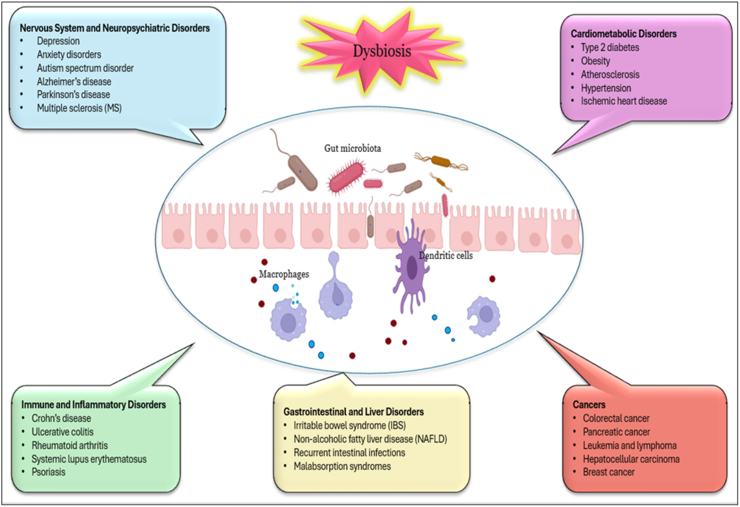
List of major diseases associated with dysbiosis.

Moreover, dysbiosis has been associated with the development and progression of certain cancers, including CRC and leukemia. It seems that specific environmental profiles play a role in the impact on the escape of inflammatory and immune cells, DNA damage, and the promotion of tumor growth and metastasis [97]. Microbial metabolites, immune signaling, and systemic inflammation are key mediators linking dysbiosis to these disease processes (Fig. 4) [2]. Recovery of microbial balance may also improve treatment outcomes. Dysbiosis affects the effectiveness of cancer immunotherapy, such as immune checkpoint inhibitors.

## 4 Mechanisms linking microbiota to HMs

### 4.1 Immune modulation: how microbiota influences innate and adaptive immune responses in cancer

The gut microbiome is increasingly recognized as a key player in blood cancers, directly influencing core features of cancer, including tumor-associated inflammation, immune evasion, genomic instability, and resistance to treatment [98]. Several studies have demonstrated that the interaction between the gut microbiota and the host is essential for the development of both innate and adaptive immunity, particularly in supporting the function and maturation of immune cells, including macrophages, B cells, T cells, neutrophils, and dendritic cells (DCs) [63]. A marked deficiency in secretory immunoglobulin A (sIgA) was the first immunological defect observed in the intestines of germ-free (GF) mice, highlighting the necessity of intestinal microorganisms for proper regulation of host immunity [63].

**Fig. 5:**
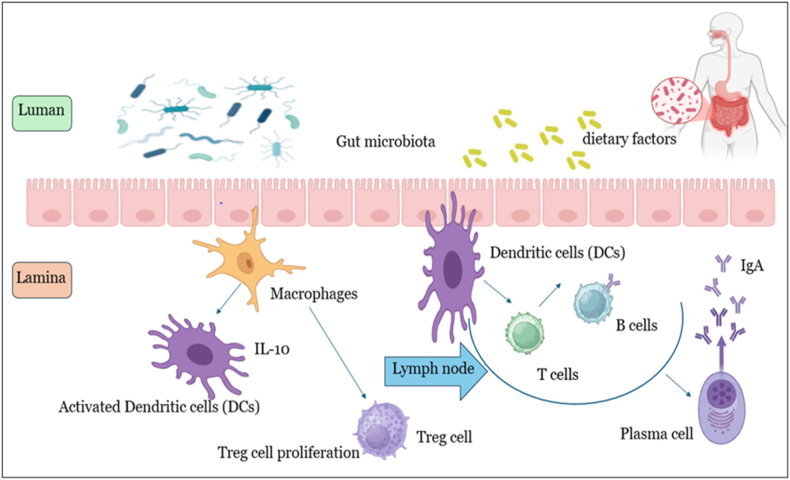
**Interaction between gut microbiota and the immune system.** How do Treg-cells, dendritic cells, and macrophages interact with gut microbiota metabolite compounds.?.

The primary antigen load of the gastrointestinal tract is composed of dietary components and gut bacterial metabolites. Metabolic compounds, including SCFAs, activate T regulatory (Treg) cells and excite macrophages and DCs. DCs are activated through two pathways, both originating from metabolites produced by intestinal bacteria and macrophages. Also, IgA antibodies are produced when B cells are activated by follicular T-cells (Fig. 5) [99].

Innate immunity is the first line of defense against cancer development and is primarily influenced by the human microbiota. Symbiotic microorganisms' impact on innate immune cells' activation, including natural killer cells and macrophages, and on the integrity of the epithelial barrier. For example, microbial metabolites, such as secondary bile acids and SCFAs, may alter cytokine production. Thus, this results in excessive inflammation and increasing anti-tumor immune surveillance [100]. Disruption of this balance (Dysbiosis) may disturb the innate immune response, lead to persistent inflammation, and reinforce tumor growth [101]. Antigen-presenting cells (APCs), such as DCs and macrophages located in the lamina propria, interpret microbial signals to maintain intestinal homeostasis and modulate adaptive immune responses [63]. The microbiota has a significant impact on modulating adaptive immunity, especially in the tumor microenvironment. Symbiotic bacteria influence the immunomodulation of effective T cells to Tregs. Effective practical function requires Th1 responses and cytotoxic T lymphocytes (CTLs) created by specific microbial species [102]. Conversely, dysbiosis can lead to the overexpansion of myeloid-derived suppressor cells and Tregs, thereby reducing immune surveillance and promoting tumor development. The gut microbiota also influences systemic immune responses, thereby modulating the effectiveness of systemic therapies, including chemotherapy and immunotherapy [103].

Microbiota-mediated increases in antitumor immunity depend on specific populations of immune cells, including DCs. Some symbiotic bacteria can enhance the function of CD8^+^ T cells and their ability to kill target tumor cells. It has been shown that the gut microbiome modulates antitumor immune surveillance and can reinforce responses to cancer immunotherapy. Specific microbial markers have been associated with responses to immune checkpoint blockade in patients with melanoma, lung cancer, and renal cell cancer. It seems that the microbiome influences systemic and antitumor immunity by educating and maturing the host immune system and by producing metabolites that can directly affect cells [101]. Recent research has also revealed the role of the microbiota in regulating responses to cancer immunotherapies. Patients with a diverse gut microbiome rich in beneficial species, such as *Bifidobacterium longum* or *Akkermansia muciniphila*, tend to have better outcomes with immune checkpoint inhibitors, including those that block CTLA-4 and PD-1 [103]. It seems that therapeutic efficacy can be enhanced by microbial metabolites and signaling pathways that improve antigen presentation and T cell recruitment in malignancies. Significantly, therapy failure has been associated with antibiotic-induced alterations in the intestinal microbiota, highlighting the importance of microbiome integrity. The microbiota can modulate innate and adaptive immunity through several mechanisms that are important for hematological oncology:

Education of DC and macrophages, modulation of the Treg/Th17 ratio, changes in the activation of NK cells and T lymphocytes that affect tumor immune surveillance, and impacts on systemic inflammatory markers. In studies of leukemias and lymphomas, different microbial patterns have been shown to correlate with disease state and response to Refs. [10,104,105].

Recent research indicates that the gut microbiota plays a role in regulating normal hematopoiesis [106,107]. Changes in the bacterial community caused by Prolonged antibiotic use, IBD, or aging have been linked to disturbances in blood cell formation [[108], [109], [110], [111]]. For example, the pretreatment microbial composition may predict the Possibility of infection in non-marrow transplantation or the response to immunotherapy [10,104,105]. Therefore, understanding how the microbiome affects both steady-state and abnormal hematopoiesis may help in developing new therapeutic strategies for blood disorders and hematologic cancers [14]. Historically, prolonged use of beta-lactam antibiotics for more than 10 days has been reported to cause severe neutropenia in humans, pointing to a link between gut bacteria and blood cell production [14]. Different antibiotic therapies have also been linked to blood-related complications, including neutropenia, anemia, thrombocytopenia, and pancytopenia [[112], [113], [114]]. Experimental studies in mice provide further evidence that the microbiota directly affects hematopoiesis. For example, oral administration of kanamycin reduced granulocyte production in the BM, indicating that intestinal bacteria are essential for maintaining myelopoiesis [115]. Similarly, mice raised under specific pathogen-free conditions and treated with broad-spectrum antibiotics developed BM suppression, which was reversible by FMT from untreated mice [110]. Furthermore, the absence of gut bacteria in antibiotic-treated mice disrupted lymphocyte homeostasis under steady-state conditions and impaired both lymphoid and myeloid differentiation, as well as immune recovery and hematopoietic reconstitution following BM transplantation [116].

The GF mice lacking a microbiota, compared with SPF mice with a healthy microbiome, exhibited abnormal BM cell populations, altered hematopoietic stem and progenitor cells (HSPCs), and dysfunctional T-cell populations [110,117,118]. Lee and colleagues demonstrated that molecules derived from the microbiota, including bacterial DNA, can travel through the systemic circulation to the BM, where they are detected by mononuclear cells and help regulate hematopoiesis under normal physiological conditions [119].

Studies have demonstrated that microbiota-derived molecules can enhance the expansion of hematopoietic progenitor cells and influence their differentiation toward myeloid lineages by inducing inflammatory cytokines such as TNFα, IL-1β, and IL-6. Inhibition of TLR signaling in CX3CR1^+^ multinucleated cells abolishes these microbiota-mediated effects on hematopoiesis. Józsefsdottir et al. reported that the effects of antibiotics on BM mesenchymal stem cells (MSCs), HSCs, and granulocytes in mice were replicated in STAT1-deficient animals, suggesting that antibiotics primarily act through microbiome depletion and disruption of STAT1 signaling, leading to impaired T-cell homeostasis and granulocyte maturation [107,110].

Recent findings indicate that microbiota-derived lactate stimulates MSCs to produce stem cell factor (SCF), thereby enhancing hematopoiesis. Mice lacking the lactate receptor Gpr81 exhibit reduced HSC numbers, and both SCF production and BM regeneration after radiation or busulfan treatment rely on Gpr81-lactate signaling. Oral administration of lactate or lactic acid-producing bacteria increases SCF secretion from leptin receptor-positive MSCs, promoting HSC proliferation and self-renewal, even in GF mice. Additionally, other microbial metabolites, such as phosphatidylcholine and gamma-glutamyl alanine, can induce type I interferon responses and mitigate hematopoietic defects caused by dysbiosis [107].

Moreover, antibiotic-induced depletion of the gut microbiota disrupts hematopoietic reconstitution after BM transplantation (BMT). It reduces absorption in mice, highlighting the essential role of the microbiota, microbial metabolites, and microbiota-dependent dietary energy in maintaining normal hematopoiesis and supporting post-transplant recovery [116].

TLR signaling within HSPCs plays a critical role in cell proliferation, cytokine production, and myelopoiesis. Activation of TLR2 or TLR4 promotes HSC proliferation and myeloid differentiation; for instance, LPS stimulates myeloid progenitors to generate macrophages, DC, and lymphoid cells [120]. Other microbial components, including beta-glucan from *Candida albicans*, CpG DNA, and Bacillus Calmette–Guérin (BCG) vaccine, have also been shown to induce HSPC differentiation and enhance myelopoiesis in a TLR-dependent manner [66].

Proinflammatory cytokines such as IL-6 and IL-1 further regulate myelopoiesis during infection or inflammation. While IL-6 is not essential for basal hematopoietic homeostasis, it contributes to inflammation-induced myelopoiesis, and IL-1 activates myeloid cell production in response to infection or inflammatory stimuli [121,122].

In summary, microbial components are essential for initiating TLR signaling, regulating HSPC proliferation, cytokine production, and myeloid differentiation, thereby supporting effective infection control. However, prolonged TLR activation resulting from chronic infection or dysbiosis may be detrimental, impairing function [14].

### 4.2 Dysbiosis and pathogenesis: role of microbial imbalances in tumor initiation and progression

One of the leading causes of carcinogenesis is microbial dysbiosis, a disruption of the balanced microbiota. In healthy individuals, symbiotic microorganisms produce metabolites, including SCFA, that have antitumor effects, control inflammation, and maintain epithelial integrity. A microenvironment appropriate for tumor initiation can be created by dysbiosis, which can decrease beneficial species and increase proinflammatory or genotoxic species [100]. Dysbiosis of the gut microbiota, characterized by a reduction in beneficial bacteria and an increase in proinflammatory and procarcinogenic species, plays a critical role in the pathogenesis of CRC [42,123]. This microbial imbalance promotes chronic inflammation and impairs immune function, thereby facilitating tumor initiation and progression [42,124]. Dysbiosis not only alters the local tumor microenvironment within the gut but can also have systemic effects, influencing the development of cancers outside the gastrointestinal tract [42,123]. One of the main mechanisms linking dysbiosis and carcinogenesis is chronic inflammation. By activating Toll-like receptors, microbial compounds such as LPS can maintain low-grade inflammation and promote mutations through oxidative stress. Early tumor formation may be initiated by this inflammatory environment, which also disrupts cellular homeostasis [101]. Microbial imbalance actively influences tumor growth and metastasis once they have begun. Signaling pathways, including NF-κB and STAT3, can be modulated by dysbiotic microbiota, which causes angiogenesis, proliferation, and resistance to apoptosis [125].

Dysbiosis affects the tumor microenvironment (TME). Certain microorganisms contribute to an immunosuppressive environment by recruiting Treg cells and myeloid-derived suppressor cells, which diminishes antitumor immune responses. These changes not only accelerate tumor growth but also affect treatment outcomes. According to recent studies, immune checkpoint inhibitors work better in people with a healthy, diverse gut microbiota, whereas dysbiosis is associated with a worse response and increased toxicity [103].

In addition to the immunomodulatory effect, several mechanisms link dysbiosis to carcinogenesis. These mechanisms include the production of genotoxic metabolites by certain bacteria that can directly damage host DNA, changes in host cell signaling pathways that control proliferation and apoptosis, and the intestinal barrier breakdown leading to systemic inflammation [123,124,126]. Induction of chronic inflammation or alterations in host metabolism create a favorable environment for tumor initiation or progression [10,75,90]. For example, some pathogenic bacteria can promote tumorigenesis by modulating the host's inflammatory response, thereby creating a protumorigenic environment [123]. Both genetic and environmental factors, including diet, antibiotic use, physical activity, aging, and obesity, contribute to this microbial imbalance [42,124]. In the field of hematological diseases, data show that chronic changes in the gut microbiome may create an inflammatory microenvironment that facilitates both carcinogenesis and survival of malignant clones. However, direct causal evidence is still accumulating, and new preclinical and epidemiological studies (2023–2025) are underway [10,75,90].

### 4.3 Metabolic interactions: microbial metabolites and their impact on cancer biology

Numerous metabolites produced by the gut microbiota are key factors that significantly affect the initiation and progression of cancer, including CRC [127].

Among microbial metabolites, SCFAs such as butyrate and propionate, tryptophan derivatives, secondary bile acids, and amino acid-derived compounds play key roles in modulating cell growth, apoptosis, inflammation, and immune responses [74,128]. SCFAs, in particular, exert protective effects by regulating immune function, maintaining epithelial barrier integrity, and promoting apoptosis in cancer cells [127].

On the other hand, dysbiosis may lead to reduced synthesis, which thereby endangers defenses. In particular, butyrate modulates epigenetic control of tumor suppressor genes by acting as a histone deacetylase (HDAC) inhibitor, thereby affecting gene expression and enhancing tumor growth inhibition [127,129], and, in some fields, it has a role as an anti-inflammatory and epigenetic protective agent [130]. However, in other fields, secondary bile metabolites or products of protein metabolism may not have tumor-protective properties [74,128]. These metabolites can act as metabolic substrates after entering the circulation and/or as immunomodulatory agents, stimulating the production of inflammatory cytokines and thereby influencing the immune response. [126,131]. In addition to their systemic effects, microbial metabolites actively influence the tumor microenvironment (TME). Some metabolites act as signaling molecules that alter the polarization and recruitment of immune cells. For example, SCFAs promote the development of anti-inflammatory Treg cells, which may reduce chronic inflammation, but may also create an immunosuppressive environment conducive to tumor growth [129]. Emerging studies also highlight the role of metabolites in response to cancer therapy. Immuno-checkpoint inhibitors often work better in patients with a diverse microbiota that produces more beneficial metabolites. On the other hand, tumor growth and resistance to therapy have been linked to the accumulation of harmful microbial products such as hydrogen sulfide and polyamines [132].

Recently, studies have focused on determining which metabolites directly play a role in HMs (e.g., lymphoma, leukemia) and have shown that metabolic manipulation (via diet, probiotics/prebiotics, floratherapy) may have potential applications as adjuvant therapy [74,128].

Table (1) illustrates the pathways and mechanisms of gut microbiota-derived metabolites and their roles in leukemia. These metabolites include compounds, LPS, amino acids, formic acid, glutamine, SCFAs, and lipids, each originating from different sources. Butyrate acts as an HDAC inhibitor, strengthening the intestinal barrier and playing a protective role against AML progression, whereas LPS activates TLR4 inflammatory pathways and exacerbates the disease. Alterations in amino acid and lipid metabolism are also associated with changes in leukemia cell nutrition and increased cell survival. Furthermore, formic acid and glutamine may serve as potential biomarkers or energy sources for cancer cells. Overall, these findings indicate that microbiota-derived metabolites can play a crucial role in the development or suppression of leukemia by modulating metabolism, immunity, and inflammation (Table 2) [7,61,62,[133], [134], [135]].

**Table 2:** Gut microbiota-derived metabolites and their pathways and mechanisms in leukemia.

Metabolite	Microbial Source	Proposed Mechanism	Effect on Leukemia	Reference
Butyrate	*Faecalibacterium* spp.	HDAC inhibitor enhances the intestinal barrier	Reduced levels associated with AML progression; supplementation delays disease	(7)
Lipopolysaccharides (LPS)	Leaked from the gut due to barrier disruption	Activates TLR4 inflammatory pathways	Elevated levels negatively affect AML progression	(7)
Amino acids & amino acid metabolites	Altered gut microbiome in AML patients	Modulates cellular metabolism and leukemia colony nutrition	Significant alterations in the fecal metabolome	(61)
Formic acid	Microbial metabolite	Accumulates in cancers	Potential biomarker for leukemia	(133)
Glutamine/Ammonia	*Klebsiella pneumoniae*, a nitrogen-fixing bacterium	Fuel for cancer cells (observed in MM)	May support leukemia cell growth	(134)
SCFAs (short-chain fatty acids)	Gut bacteria	Modulate immunity, cellular metabolism, tumor microenvironment	Potential role in leukemia progression	(135)
Lipid & sphingolipid pathways	Altered lipid metabolism	Affects leukemia cell survival and proliferation	Dysregulated lipid metabolites observed in ALL/CLL	(62)

#### 4.3.1 Differential roles of butyrate across hematological malignancy subtypes

While the role of butyrate as an HDAC inhibitor is introduced generally in the preceding section, its mechanistic relevance differs substantially across hematological malignancy subtypes and cannot be treated as a uniform class effect. Three distinct mechanistic contexts are relevant to the diseases covered in this review.

##### 4.3.1.1 Butyrate in AML: HDAC inhibition and epigenetic tumor suppressor reactivation

In AML, the mechanistic case for butyrate is best established and most directly supported by clinical data. AML cells characteristically exhibit aberrant HDAC activity, which silences tumor suppressor genes including p21, p27, and CDKN1A via histone deacetylation. Butyrate, produced by *F. prausnitzii* and related *Firmicutes*, inhibits class I and II HDACs by competitive occupancy of the zinc-containing catalytic site, leading to histone hyperacetylation and re-expression of silenced tumor suppressor loci. Wang et al. (2022) demonstrated in AML patient-derived samples and murine models that reduced luminal butyrate concentrations correlate with accelerated AML progression, and that butyrate supplementation delays leukemic expansion by restoring CDKN1A expression and inducing G1 cell cycle arrest. This HDAC-dependent mechanism is therefore directly relevant to AML biology and represents a tractable therapeutic target for SCFA-augmenting dietary and probiotic interventions [136].

##### 4.3.1.2 Butyrate in CLL: BCL2-Dependent apoptosis and HDAC1-Mediated transcriptional dependencies

In CLL, the mechanistic role of butyrate differs from that in AML and operates through a distinct set of molecular dependencies. CLL cells are characterized by constitutively elevated BCL2 expression, a direct transcriptional consequence of high HDAC1 activity, which establishes epigenetic dependencies that sustain CLL cell survival by suppressing pro-apoptotic gene expression. Multiple studies have demonstrated that HDAC1 enzymatic activity is elevated in CLL and positively correlates with poor overall survival, and that the BCL2 promoter is maintained in a deacetylated, transcriptionally active state by HDAC1-mediated chromatin remodeling.

Butyrate, by inhibiting HDAC1 activity in CLL cells, disrupts this dependency in two complementary ways: it promotes histone hyperacetylation at the BCL2 promoter (paradoxically leading to chromatin remodeling that reduces BCL2 transcriptional output via altered enhancer-promoter interactions). It simultaneously induces the expression of pro-apoptotic BH3-only proteins, including BIM and NOXA, shifting the BCL2/BAX rheostat toward apoptosis. Sodium butyrate has been shown to induce caspase-3 and caspase-9 activation via the intrinsic mitochondrial apoptotic pathway in lymphoid malignancy cells, with downstream BCL2 downregulation and BAX upregulation. This mechanistic pathway is particularly clinically relevant in the context of venetoclax (BCL2 inhibitor) therapy in CLL: dysbiosis-driven depletion of butyrate-producing organisms may, by sustaining HDAC1 activity and BCL2 overexpression, contribute to a microbiota-mediated mechanism of venetoclax resistance. Conversely, restoring luminal butyrate through probiotic or dietary intervention could sensitize CLL cells to BCL2-targeted therapy, a hypothesis that remains untested in prospective clinical trials but is mechanistically grounded and represents a priority for translational investigation [137].

##### 4.3.1.3 Butyrate in MM: Th17 suppression and IL-17/IL-6 axis modulation

In MM, butyrate's most disease-relevant mechanism operates through the gut-BM immune axis described in Section 2.1.7. Butyrate promotes colonic Treg differentiation and suppresses Th17 cell development by inhibiting RORgammat transcriptional activity, the master regulator of Th17 lineage commitment. Since Th17 cells are the proximate effectors of the *P. heparinolytica*-driven BM inflammatory cascade that accelerates progression from smoldering to symptomatic MM, butyrate depletion removes a critical brake on this pathological axis. Reduced butyrate, therefore, in MM patients does not merely reflect generalized dysbiosis; it actively licenses gut-to-bone marrow Th17 migration that drives IL-17 production, eosinophil activation, and IL-6-mediated plasma cell proliferation. Restoration of butyrate levels through SCFA-producing probiotic supplementation or dietary fiber enrichment may therefore suppress this disease-specific circuit in a mechanistically targeted manner [63,138,139].

#### 4.3.2 LPS in MM: MAPK-driven plasma cell proliferation distinct from AML TLR4-NF-kB signaling

The current manuscript describes LPS as activating TLR4 inflammatory pathways in leukemia in a generalized manner. However, LPS-mediated signaling in MM operates through a mechanistically distinct intracellular pathway compared to its effects in myeloid leukemias, and this distinction has direct implications for understanding disease-specific pathogenesis and therapeutic targeting.

In AML and other myeloid malignancies, LPS acts primarily through the canonical TLR4-MyD88-NF-kB signaling axis, inducing proinflammatory cytokine production (TNF-α, IL-1β, IL-6) that creates a permissive inflammatory microenvironment for leukemic clone expansion. This pathway is well established and described in the existing manuscript text. In MM, however, LPS directly stimulates plasma cell proliferation via a parallel and partly distinct intracellular cascade: the MAPK/ERK (mitogen-activated protein kinase/extracellular signal-regulated kinase) pathway. In vitro studies using IL-6-dependent murine plasmacytoma and hybridoma cell lines validated models of human MM biology. It demonstrated that LPS-induced plasma cell proliferation is substantially blocked by the selective MEK inhibitor PD98059, whereas IL-6-induced proliferation in the same cell lines proceeds through STAT3 activation, not MAPK. This establishes a TLR4-MAPK-ERK axis as a parallel pro-proliferative signal in MM, independent of and additive to the canonical IL-6-STAT3 pathway, which currently dominates MM therapeutic targeting.

The gut microbiota relevance is direct: barrier disruption from MM-associated dysbiosis (itself driven by SCFA depletion, nitrogen-recycling bacteria, and Th17-mediated BM inflammation) increases systemic LPS translocation. Elevated circulating LPS then reaches the BM microenvironment, where it activates TLR4 on both myeloma plasma cells (via MAPK/ERK) and BM stromal cells (via NF-kB, inducing paracrine IL-6). The two pathways converge on plasma cell proliferation and anti-apoptotic signaling: MAPK/ERK promotes cyclin D1 expression and G1 cell cycle entry, while IL-6/STAT3 sustains BCL2 and MCL1 expression and resists apoptosis. This mechanistic duality, LPS stimulating both autocrine MAPK proliferation in myeloma cells and paracrine NF-kB-IL-6 production in stromal cells, makes gut-derived endotoxemia a particularly potent driver of MM progression that is not fully captured by describing LPS effects generically as inflammatory [[140], [141], [142], [143], [144], [145]].

## 5 Microbiota and treatment outcomes

### 5.1 Chemotherapy and immunotherapy: influence of microbiota on treatment efficacy and toxicity

The microbiota is now recognized as a key component of human pathophysiology. Depending on its composition and structure, it can either protect against certain diseases or contribute to their development [146]. A good example of this link is that some types of vaginal community state type (CST) related to cervical cancer or the presence of *Fusobacterium nucleatum* are often found in cases of CRC [147]. It seems that chemotherapy is affected by efficacy mechanisms and toxicity. So far, the choice of chemotherapy regimen, its dose, route of administration, and other treatment features have mainly followed established protocols. These protocols rely on the pharmacokinetic and pharmacodynamic behavior of the drugs used and are adjusted according to each patient's condition, including their disease type, clinical status, and body characteristics [148]. Chemotherapy is known to frequently induce a broad spectrum of adverse drug reactions (ADRs), with gastrointestinal toxicity being ubiquitous in treatment with BCR-ABL and EGFR inhibitors, presenting as diarrhea, vomiting, nausea, and abdominal pain [149].

The gut microbiome also plays a crucial role in shaping the body's response to chemotherapy and immunotherapy [150]. Changes in the composition, diversity, and dynamics of microbial communities can markedly influence the effectiveness of these treatments. Mechanisms of this influence include modulating drug metabolism, regulating inflammatory pathways, and stimulating or inhibiting innate and adaptive immune responses [150]. Findings from clinical research indicate that the makeup of the microbiota can shape how the body responds to cancer treatments. Adjusting or supporting the microbial balance may help strengthen the drugs' action and ease their side effects. It is also evident that both endogenous and introduced microorganisms can alter the course of chemotherapy, demonstrating that the connection between microbes and cancer therapy operates in both directions [151].

In more detail, various gut microbial communities can alter how patients respond to chemotherapy, either enhancing or reducing its therapeutic effect. Conversely, chemotherapy itself may disturb the intestinal flora, leading to an imbalance (dysbiosis) and local tissue damage that makes the gut more vulnerable to drug-related toxicity, such as mucositis [152].

The microbiome affects the outcomes of chemotherapy through multiple mechanisms:

The first mechanism is the modification of the response to chemotherapy by the microbiota, through the induction of chem. The microbiota influences membrane pump function and regulates cytotoxins, thereby affecting the amount of drug excreted, the drug dose remaining inside the tumor cell, and, consequently, treatment potency [153]. Another relevant mechanism involves the compensatory response of deoxyribonucleic acid (DNA) that can be influenced by the microbiota [154]. However, one of the most significant processes linked to chemoresistance appears to be the ability of certain bacterial species to interact with chemotherapeutic agents-by modifying or metabolizing them, which in turn alters their treatment effectiveness [155]. Chemotherapy is frequently required for patients with leukemia; however, it can disrupt microbiota and compromise the protective function of the intestinal barrier. For example, A severe side effect of Chemotherapy is mucosal injury and gastrointestinal ulceration in patients with HL [156]. This disruption can affect the chemotherapy efficiency [157], as chemotherapy often disrupts the delicate balance between beneficial and harmful bacteria [156].

Gut microbes can influence the efficacy and toxicity of chemotherapy through transport, immune modulation, metabolism, and enzymatic degradation; alterations in the tumor microenvironment and metabolic changes [158]. Gut microbes can change drug efficacy by metabolizing drugs, changing drug concentrations, and activating or inhibiting the immune system [150]. One mechanism is that some bacteria can metabolize or alter the pharmacokinetics of therapeutic drugs, thereby altering their activity. For example, *Enterococcus faecalis* and *E. coli* can reactivate the drug irinotecan via β-glucuronidase enzymes, leading to increased gastrointestinal toxicity [159]. In contrast, some species, such as *Lactobacillus rhamnosus* and *B. longum*, modulate chemotherapy side effects by reducing inflammation and maintaining mucosal barrier function. [160]. For example, gut commensal bacteria can directly or indirectly influence the efficacy of various chemotherapeutic agents, such as cyclophosphamide, oxaliplatin, and irinotecan [10]. Disruption of the gut microbiota may stimulate inflammatory responses, affect cell and immune system function, and finally weaken immunity [161]. For instance, in a phase/clinical study, administering autologous FMT to a small number of AML patients who had received chemotherapy and antibiotics helped restore gut microbial diversity and reduced intestinal inflammation markers, such as C-reactive protein [14]. Therefore, preserving a balanced gut microbiota in patients undergoing chemotherapy is essential to enhance effectiveness and reduce toxicity. It has been shown that Probiotics supplementation or probiotic drugs improve chemotherapy efficacy and decrease side effects [162]. Cyclophosphamide is a standard chemotherapy drug used for HMs, neuroblastoma, some sarcomas, and breast and ovarian cancers [163]. Its antitumor activity is broad and partially comes from its ability to boost the immune response against tumors [164]. Recent studies have confirmed that, once given, it can favor the growth of specific bacterial groups in lymphoid tissues, leading to greater production of immune cells that target tumor antigens [165,166]. The drug shows decreased efficacy when coadministered with antibiotics targeting Gram-positive bacteria, suggesting that bacteria such as *Enterococcus* and *Lactobacillus* enhance CP-mediated antitumor responses by modulating Th1 and Th17 cells [166,167]. Probiotics can also reduce oral mucositis and improve oral health by increasing the population of protective bacteria, thereby decreasing treatment-related side effects [168]. Chronic gastrointestinal inflammation and chemotherapy-induced diarrhea can be decreased with probiotics [162]. 5-Fluorouracil (5-FU) is widely used for the treatment of malignant tumors, but it can cause gastrointestinal side effects by inducing apoptosis, inhibiting enterocyte proliferation via TNF-α, weakening epithelial barrier function, and causing inflammation. Combination therapy with broad-spectrum antimicrobials, such as ampicillin and aztreonam, can decrease the severity of nasal mucositis. Probiotics administered with chemotherapeutic agents increase antitumor responses. For example, *Lactobacillus acidophilus* with cisplatin in mice with lung cancer decreased tumor volume and increased survival by upregulating IFN-γ and Prf 1 [169]. Similarly, a probiotic mixture containing strains of *Lactobacillus lactis* and *Bifidobacterium bifidum* improved the efficacy of 5-FU in CRC models [170]. The chemotherapeutic drug irinotecan affects the gut microbiota by interacting with microbial enzymes and modulating the immune system, thereby influencing both its efficacy and toxicity[5]. Specific bacterial species, such as *Lactobacillus johnsonii* and *Enterococcus, Shigella*, can translocate to the spleen, activate Th1 and Th17 immune responses, and enhance the effectiveness of cyclophosphamide [150]. These findings underscore the potential of microbiota-targeted interventions as a form of personalized cancer therapy, although further studies are needed to validate their benefits in leukemia models. The gut microbiome exerts a profound influence on host immunity [135].

CpG oligodeoxynucleotides consist of unmethylated CG dinucleotides that act as immunostimulatory agents and can potentiate the effectiveness of anticancer antineoplastic treatments [171]. The activity of CpG oligodeoxynucleotides is also strongly modulated by the gut microbiome. For example, CpG treatment in EL4-bearing mice showed reduced efficacy compared to antibiotic-treated or GF mice [165]. Additionally, the composition of the gut microbiome has been shown to affect the response to immune checkpoint inhibitors (ICIs) in various cancers, including melanoma, lung cancer, and renal cell carcinoma [172]. Patients with higher abundances of specific bacterial species, such as *A. muciniphila*, *B. longum*, and *F. prausnitzii*, often show better clinical responses to ICI treatment [173]. Conversely, antibiotic use that disrupts the gut microbiota has been associated with more weakness in cancer patients treated with ICI [172].

Overall, evidence suggests that a healthy and diverse microbiota can enhance response to anticancer therapies, while dysbiosis caused by drugs, infections, or other factors may reduce treatment efficacy and increase toxicity. These findings provide a strong basis for developing microbiome-based interventions to enhance the efficacy of anticancer therapies.

Table 3 examines the role of microbial metabolites in hematological cancers and their relationship with immune responses and diseases. This table lists several key metabolites produced by the gut microbiome, including SCFAs, I-P, and TMAO. Additionally, the proposed mechanisms related to the effects of these metabolites on hematological cancers and other diseases are discussed. SCFAs are emphasized for their role in regulating immunity and gut health, particularly in patients with AML, while the effects of the metabolite TMAO on altering microbiota and their interactions with the immune system and inflammation are also highlighted. SCFAs can improve patients' symptoms of painful inflammation and boost immune cell function. On the other hand, TMAO serves as a metabolic marker that can be valuable for analyzing microbiome profiles and identifying functional abnormalities (Table 3) [10,61,135,174].

**Table 3:** The role of microbial metabolites in hematologic malignancies and their relationship with immune responses and disease.

Metabolite/Example	Microbial Source/Production Pathway	Proposed Mechanism Related to Hematologic Cancers/Therapies	Key Study Evidence	Reference
Short-chain fatty acids (SCFAs), Butyrate	Produced by *Firmicutes* species in the gut	Immune and epigenetic regulation of immune cells, associated with therapy response in AML	Changes in SCFAs observed in AML patients	(61,135)
Indole derivatives (IPA and IAA)	Derived from tryptophan metabolism by gut microbes	Activation of AhR receptor and strengthening of the gut barrier; reduction of inflammation and GVHD in transplant models	Preclinical data in GVHD models	(10,135)
Trimethylamine-N-oxide (TMAO)	Produced from choline/carnitine metabolism by microbes, converted to TMAO in the liver	Macrophage (M1) polarization and NLRP3 activation; exacerbates GVHD in BM transplant models	Animal and clinical studies	(10,174)
Reduced butyrate and IPA under benzene exposure	Altered microbial composition and fatty acid/indole metabolite pathways	Reduction of protective metabolites leads to hematopoietic damage in animal models	Animal model of benzene toxicity	(175)
Tryptophan–kynurenine pathway	Shared microbial-host pathway via IDO/TDO enzymes	Increased immunosuppressive kynurenine metabolites suppress antitumor responses and alter the immune microenvironment.	Reviews on cancer and immunity	(135)

#### 5.1.1 Microbiota and CAR-T cell therapy: disease-specific evidence in HMs

Chimeric antigen receptor T cell therapy has dramatically reshaped the management of relapsed and refractory hematological malignancies. Currently, six products approved by both the FDA and EMA are available for the treatment of B-cell non-Hodgkin lymphoma, B-cell acute lymphoblastic leukemia, and multiple myeloma. Although these therapies can induce remarkable initial responses, long-term outcomes remain limited by disease recurrence and treatment-related toxicities. Clinical studies indicate that relapse occurs in a substantial proportion of patients, while adverse events such as cytokine release syndrome and immune effector cell-associated neurotoxicity syndrome affect a large percentage of treated individuals. Emerging evidence suggests that the intestinal microbiome plays an important role in regulating both therapeutic efficacy and toxicity following CAR T cell therapy. This interaction has become increasingly relevant in HMs and represents an important area of investigation for improving treatment outcomes [30,176].

##### 5.1.1.1 Antibiotic-induced dysbiosis and CAR-T outcomes: the P-I-M signal

Among the microbiome-related factors that influence CAR T cell therapy, exposure to broad-spectrum antibiotics before treatment appears to be one of the most clinically significant. Smith and colleagues conducted a multicenter retrospective study involving 228 patients with B-cell lymphoma and leukemia who received anti-CD19 CAR T cell therapy. Their findings demonstrated that treatment with piperacillin-tazobactam, imipenem-cilastatin, or meropenem during the 4 weeks preceding CAR T infusion was associated with poorer overall survival and a higher incidence of neurotoxicity. These antibiotics possess strong activity against anaerobic bacteria and can profoundly alter gut microbial communities.

The same study also included a prospective cohort of 48 CAR T recipients whose fecal microbiota were analyzed before infusion. Compared with healthy individuals, patients exhibited markedly reduced microbial diversity, increased dominance of individual bacterial taxa, and an overall microbial composition that differed substantially from that of healthy controls. Importantly, a higher abundance of *Ruminococcus* was associated with complete remission at day 100 after therapy, whereas greater levels of Bacteroides correlated with lower toxicity rates. These observations indicate that the gut microbiome present at the time of CAR T infusion actively influences therapeutic response and adverse event profiles rather than simply reflecting the patient's clinical condition [30].

Stein Thoeringer and colleagues later confirmed these observations in an independent multicenter cohort comprising 172 patients with B-cell lymphoma treated with CD19-targeted CAR T cells across Germany and the United States. Among patients who had not received high-risk antibiotic regimens before treatment, a greater abundance of *Bifidobacterium longum* and enhanced microbial capacity for peptidoglycan biosynthesis were significantly associated with improved six-month survival [176].

These findings suggest a potential mechanistic explanation: microbiome-derived peptidoglycan promotes innate immune activation and enhances antigen cross-presentation, thereby strengthening the antitumor activity of CAR T cells against hematological cancers.

##### 5.1.1.2 Disease-specific microbiome-car-T interactions: MM, ALL, and NHL

The most detailed evidence regarding disease-specific microbiome interactions during CAR T therapy comes from the prospective study conducted by Hu et al. (2022). This investigation followed changes in gut microbial composition throughout different stages of CAR T treatment in patients with relapsed or refractory MM, B-cell ALL, and NHL [177].

• MM (BCMA CAR-T): In patients with MM, those who achieved complete remission displayed significantly greater abundance of Bifidobacterium, Prevotella, Sutterella, and Collinsella than patients who achieved only partial remission. The composition of the intestinal microbiome also changed over time, with distinct microbial profiles observed before infusion, after infusion, and during response evaluation. Interestingly, severe cytokine release syndrome was associated with elevated pre-infusion levels of Bifidobacterium, Leuconostoc, Stenotrophomonas, and Staphylococcus, suggesting that baseline microbial composition may influence inflammatory toxicity [177].
• B-ALL (CD19 CAR-T): Patients with ALL demonstrated a microbiome profile that differed substantially from those observed in multiple myeloma and NHL. These differences were characterized by patterns consistent with previously reported leukemia-associated dysbiosis, particularly enrichment of *Proteobacteria* and *Enterococcus* species. The microbial environment present at the time of CAR T infusion likely reflects the cumulative effects of previous chemotherapy regimens and repeated antibiotic exposure throughout treatment [177].
• NHL (anti-CD19 CAR-T): In both the Smith et al. and Hu et al. studies, increased abundance of *Ruminococcus, Bacteroides,* and *Faecalibacterium* was consistently associated with more favorable therapeutic outcomes following CAR T therapy in NHL. These microorganisms are known producers of SCFAs and possess important immune regulatory properties, which may explain their beneficial association with treatment efficacy [30,176,177].

##### 5.1.1.3 Proposed mechanisms: how gut microbiota modulates CAR-T efficacy

Current evidence suggests that the intestinal microbiota affects CAR T cell therapy outcomes through several interconnected biological pathways. Rather than operating independently, these mechanisms likely interact to shape both antitumor efficacy and treatment-related toxicity in patients with hematological malignancies [178,179].

• Antigen cross-presentation priming:
• One proposed mechanism involves enhancing antigen cross-presentation by microbial products. Certain bacterial metabolites and cell wall components may activate innate immune pathways, thereby improving antigen processing and presentation by dendritic cells. This process can strengthen CAR T cell activation and enhance tumor cell elimination [178].
• Modulation of Systemic Immune Tone: The gut microbiota also regulates systemic immune activity through metabolite production, cytokine modulation, and communication with immune cells. These effects may influence CAR T cell expansion, persistence, and functional activity after infusion [180].
• Amplification of Cytokine Release Syndrome (CRS) Through Lipopolysaccharide Translocation:

Another proposed mechanism involves disruption of intestinal barrier integrity, leading to the translocation of lipopolysaccharides into the systemic circulation. Increased exposure to these proinflammatory molecules may intensify cytokine production and contribute to the development or severity of cytokine release syndrome and neurotoxicity following CAR T treatment [181].

### 5.2 Stem cell transplantation: microbial contributions to transplant success and complications

Suitable reconstitution of the immune system is central to successful hematopoietic cell transplantation (HCT). Insights from neonatal immune development suggest that the microbiota and adaptive immunity rapidly develop during the first three years of life, and that disruptions in these systems lead to short- and long-term immune outcomes [182].

**Fig. 6:**
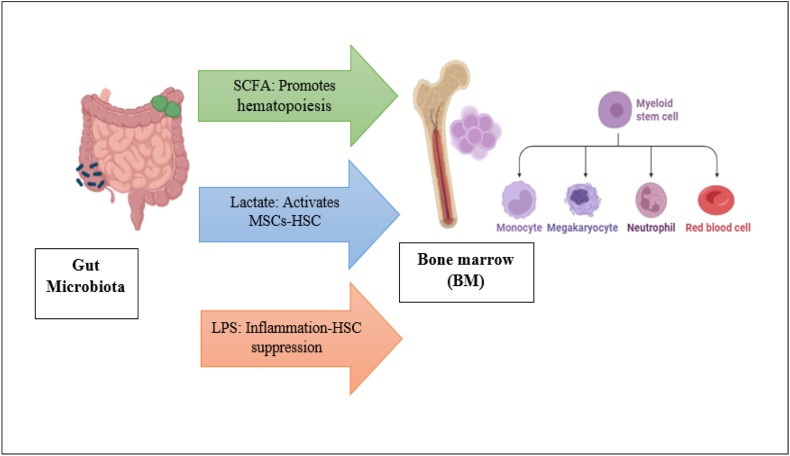
**Schematic representation of the gut-BM axis and the impact of microbial metabolites on hematopoiesis.** Beneficial microbial products, such as short-chain fatty acids (SCFAs) and lactate, promote the regeneration of hematopoietic stem cells (HSCs) and the balanced regulation of hematopoiesis, whereas lipopolysaccharide (LPS), produced by intestinal dysbiosis, induces inflammation and inhibits hematopoietic activity.

Studies using GF animal models have demonstrated that the absence of gut microbiota leads to underdeveloped mucosal immunity, including the lack of gut-associated lymphoid tissues such as Peyer's patches and mesenteric lymph nodes [183]. Consequently, in the context of allo-HCT, maintaining the integrity of the gut microbiota is crucial for patient outcomes (Fig. 6) [184]. Clinical evidence indicates that reduced gut microbiome diversity following transplantation is associated with an increased risk of graft-versus-host disease (GvHD), severe infections, and higher mortality [184,185]. Specifically, loss of obligate anaerobes is associated with a higher risk of GvHD, whereas diversity maintenance is associated with improved survival [20]. Studies conducted at major transplant centers, such as Memorial Sloan Kettering, have shown that patients with a predominance of particular species, such as *Enterococcus* or *Streptococcus*, in the first days after transplantation were most likely to develop severe GvHD. In contrast, maintaining a diverse beneficial flora, particularly the presence of Bacteroides and Blautia, has been associated with a reduced risk of GvHD and improved survival [15].

Moreover, the use of immunosuppressive drugs to prevent or manage GvHD increases patients’ susceptibility to *Clostridium difficile* infection [186]. After allogeneic hematopoietic stem cell transplants (HSCT), approximately 40% of patients have been reported to develop *C. difficile* infection after 9 months [187]. This effect may be related to reduced microbial products, including SCFAs such as butyrate and propionate, disruption of the internal epithelial barrier, and overstimulation of inflammatory pathways by pathogens. These results have led to interventions, including the use of safe probiotics, dietary adjustments, and even the pre- or post-transplant period [188]. The immunoregulatory effects of the gut microbial community have been investigated for the treatment of acute GVHD after allo-HSCT. For example, the effect demonstrated efficacy in patients with GVHD resistant to corticosteroids [189]. However, there is still no clear standard for selecting beneficial species or for determining the appropriate timing of intervention, and more randomized studies are needed.

### 5.3 Case studies and clinical evidence: key findings from human and animal studies

Clinical studies have consistently demonstrated a strong association between gut microbiome composition and outcomes following allo-HCT [190]. For instance, a key study reported that patients with higher gut microbiota diversity at the time of neutrophil engraftment experienced lower mortality, primarily due to reduced GvHD-related deaths [19]. Animal studies have been important in elucidating the mechanisms, showing that certain microbes can train the immune system and that transfer of a healthy microbiota can protect against GvHD in mouse models [10]. Recent research on HMs, particularly in the context of hematopoietic stem cell transplantation (HSCT), indicates that alterations in the gut microbiota may predict complications, including GvHD, infections, and overall mortality [[191], [192], [193]]. Decreased gut microbiota diversity after transplantation is associated with an increased risk of GVHD and mortality. Lower diversity in the early weeks after transplantation is associated with a higher risk of complications [191].

A prospective clinical study including 29 patients with AML, 17 with CML, and 33 healthy controls found that the phyla *Actinobacteria*, *Acidobacteria*, and *Chloroflexi* were relatively more abundant. In contrast, *Tenericutes* were decreased in CML patients, while AML patients exhibited increased levels of *Streptococcus* and reduced abundances of *Megamonas*, the *Lachnospiraceae* NC2004 group, and *Prevotella* 9 [48].

In another study, 16S rRNA quantitative profiling combined with bioinformatic analysis of fecal samples revealed significant microbial alterations in patients with ALL compared with healthy individuals. Notably, *B. clarus* was markedly increased in the fecal microbiota of ALL patients [38].

Gut microbes influence the development of T cells, which are crucial for immune responses after transplantation [192]. Findings from both human and animal studies have established a clear link between the composition of the microbiota and the outcomes of cancer treatments. In a clinical study conducted by Kim et al. (2024), patients with melanoma who were resistant to anti-PD-1 therapy partially regained immune responses after receiving FMT from responding donors. These results show for the first time that targeted microbiome restoration can reverse resistance to immunotherapy [194]. Nguyen et al. conducted a study involving 301 breast cancer patients treated with taxane-based chemotherapy, among whom 44.5% had undergone prior surgery. The study found that severe hematologic toxicity during chemotherapy was associated with a higher abundance of *Firmicutes* in pretreatment samples, while lower levels of *Campylobacter* correlated with an increased risk of severe hematologic toxicity [62].

More recently, in 2025, Boehm and colleagues reported growing evidence that gut microbiota composition influences chemotherapy outcomes. In lung tumors, patients who responded better showed relatively higher abundances of *Streptococcus mutans*, *Enterococcus caesaliflavus*, and *Bacteroides*, while in gastrointestinal tumors, higher levels of *Lactobacillaceae*, *Bacteroides fragilis*, and other species were associated with improved response [195].

In 2023, they found that FMT modulates the gut microbiota and can improve treatment outcomes by decreasing side effects [193].

A systematic review by Wekking et al. (2025) also included more than 40 human studies and concluded that higher microbial diversity and abundance of specific *Bifidobacterium* and *Akkermansia* species were associated with better efficacy of anticancer therapies [196].

In an innovative clinical trial, patients with melanoma who had not responded to anti-PD-1 therapy exhibited a favorable immune response in more than one-third of cases following FMT from responders (Kim et al., 2024). Favorable changes in the concentrations of anti-inflammatory metabolites and increases in *A. muciniphila* and *B. longum* species were also observed [197].

Similar evidence has been observed in preclinical studies: in animal models, antibiotic-induced dysbiosis decreased the response to immunotherapy and increased tumor growth, whereas microbiome restoration via probiotics or FMT had the opposite effect [198].

Furthermore, several animal studies have demonstrated the antitumor properties of probiotics. Administration of *Bacillus polyfermenticus*, *Bifidobacterium infantum*, *B. bifidum*, as well as *L. acidophilus*, *L. casei, L. lactis, L. plantarum, L. rhamnosus, and L. salivarius* to tumor-bearing mice significantly reduced CRC formation [150].

Moreover, while intestinal bacteria contribute to lowering carcinogenesis through the metabolism of fiber-rich diets and the generation of SCFAs such as butyrate, branched-chain amino acids, and bile acids, fasting has also been proposed as a potential approach to alleviate irinotecan-related side effects in FabplCre; Apc15lox/+ mice without compromising its anticancer activity [199].

Overall, these findings emphasize that the interaction between the gut microbiota and anticancer therapies is both mechanistically and clinically significant. A deeper understanding of these interactions could help optimize therapeutic efficacy while minimizing the adverse effects of current cancer therapies.

## 6 Therapeutic potential of microbiota modulation

### 6.1 Probiotics and prebiotics: strategies to restore microbial balance

Diet is a key factor influencing the composition of the gut microbiota, and numerous studies have investigated the relationship between dietary patterns, the microbiome, and cancer risk. A Western-style diet, which is typically high in animal fats and proteins but low in dietary fiber, has been associated with an increased risk of cancer. This is thought to occur through the accumulation of bile acids and a reduction in the microbial production of SCFAs [200]. Despite this, evidence on whether dietary interventions can enhance the effectiveness or reduce the toxicity of chemotherapy and immunotherapy remains limited. Animal studies have provided some insight into the diet–microbiome–treatment relationship. For example, diets enriched with protein sources (casein and whey), L-leucine, fish oil, and oligosaccharides have been shown to prevent *Pseudomonas aeruginosa* translocation and reverse cyclophosphamide-induced neutropenia [201].

One novel approach in complementary cancer therapies is the use of probiotics and prebiotics to restore the intestinal microbiome balance. These strategies are used to increase the number of beneficial bacteria, enhance the intestinal barrier function, and modulate inflammatory responses [202]. Modulating the microbiota may decrease gastrointestinal toxicity, the risk of infection, and support immune recovery [203].

Probiotics are live microorganisms, such as *Lactobacillus*, *Bifidobacterium*, and *Akkermansia* species, that can modify the intestinal environment, influence the intestinal metabolome, and interact with the host immune system. By producing metabolites, such as chain fatty acids, particularly butyrate, probiotics can suppress inflammatory signaling and promote Treg activation [204,205]. They may be beneficial for health, and their administration may reduce systemic inflammation, improve response to anticancer therapies, decrease treatment-related gastrointestinal toxicities such as mucositis and chemotherapy-induced diarrhea, and prevent infections [150,206]. Inhibitory pathways and activating T regulatory cells (Tregs) by producing metabolites such as SCFAs, including acetate, propionate, and butyrate. These metabolites contribute to immune homeostasis by enhancing anti-inflammatory responses, suppressing excessive immune activation, and maintaining intestinal barrier integrity [205]. In addition, probiotics use may help improve intestinal epithelial health by modulating cytokine secretion (such as IL-10 and TGF-β) and increasing the expression of mucosal adhesion proteins (such as zonula occludens) [202]. The protective role of *Lactobacillus fermentum* BR11 against 5-fluorouracil (5-FU)-induced intestinal mucositis has also been investigated, indicating that this strain can help reduce the chemotoxic effects of the drug [207].

However, the effects of probiotics depend on the strain, dose, timing of administration, and individual patient characteristics. Some strains may pose a risk of infection in immunosuppressed patients (especially after BM transplantation or severe chemotherapy) [15]. Prebiotics, on the other hand, are nondigestible dietary components that promote the growth of beneficial gut bacteria. These compounds, primarily dietary fibers, undergo fermentation by intestinal microorganisms, supporting a healthy gut environment by selectively stimulating the growth and metabolic activity of beneficial bacteria such as *Lactobacillus* and *Bifidobacterium* [208]. This process leads to the production of SCFAs, which contribute to intestinal and systemic health. These SCFAs help maintain intestinal barrier function, regulate host metabolism, and strengthen immune responses, thereby offering protection against several diseases [209]. Early clinical studies have shown that combining probiotics with prebiotic fibers (such as inulin and fructooligosaccharides) can increase microbial diversity and reduce diarrhea and opportunistic infections in cancer patients undergoing chemotherapy [202]. In children undergoing hematopoietic stem cell transplantation, *Lactobacillus* species were well tolerated and safe, even in patients with mucosal damage [210]. Symbiotic supplementation (*B. longum* with guar gum) improved gastrointestinal symptoms and mucosal integrity in patients undergoing autologous hematopoietic stem cell transplantation [211]. Animal studies combining *Lactobacillus reuteri* with inulin-type fructans showed improved intestinal barrier function, reduced inflammation, suppressed leukemia cell proliferation, and increased patient survival [212]. Prebiotics, such as a soy protein-whey blend, also accelerated hematopoiesis recovery after hematopoietic stem cell transplantation [213]. Their effects depend on strain and dose, and are sensitive to time. Safety in immunocompromised patients should be carefully evaluated due to the potential risk of blood infections [206,210,213]. However, the use of probiotics in severely immunocompromised patients remains controversial; Therefore, it is crucial to identify probiotic strains that are both safe and effective, and to conduct large-scale clinical trials to validate their safety and therapeutic potential. Postbiotics refer to mixtures of micro- and macromolecular substances, including nonviable microbial cells, cell fragments, metabolites, and by-products of probiotic activity, which can influence physiological functions when consumed in adequate quantities [214]. Because living bacteria in probiotic supplements may lose viability or activity under poor storage, processing, or environmental conditions, postbiotics are considered a more stable and safer alternative. Postbiotics are now recognized as key modulators of gut health and function, showing anti-inflammatory, antioxidant, immunomodulatory, antihypertensive, and anti-apoptotic effects.

#### 6.1.1 Strain selection: efficacy and safety are not interchangeable across species

One of the most important yet often neglected concepts in probiotic research is that the biological effects of probiotics are highly strain-specific. Consequently, findings obtained for one strain should not be extrapolated to other strains, species, or even members of the same genus. In patients with hematological malignancies, current evidence highlights three microorganisms of particular interest.

• *L. rhamnosus* GG (LGG): *L. rhamnosus* GG (LGG) is among the best-characterized probiotic strains in oncology and hematopoietic transplantation research. This strain contributes to maintaining intestinal barrier integrity by modulating TLR2 and TLR4 signaling pathways and has demonstrated beneficial effects in reducing chemotherapy-associated gastrointestinal toxicity. Evidence from several clinical studies supports its role in decreasing chemotherapy-related diarrhea. In pediatric acute leukemia, Reyna-Figueroa et al. (2019) [162] conducted a randomized pilot study involving 60 children undergoing induction or reinduction chemotherapy. Children who received oral LGG supplementation at a dose of 5 × 10^9^ CFU twice daily had significantly lower rates of nausea, vomiting, and abdominal distension than the control group. LGG is generally considered safe, but its administration in highly immunocompromised populations requires caution. Gilliam et al. (2022) [215] described six cases of *Lactobacillus* bacteremia among pediatric hematopoietic cell transplant recipients treated at St. Jude Children's Research Hospital. Whole-genome sequencing revealed genetic similarity between the bloodstream isolates and the probiotic products administered at the time, indicating that probiotic-associated bacteremia can occur even with extensively studied strains such as LGG. These findings emphasize that, although uncommon, the risk should not be considered negligible in transplant recipients.
• *B. longum*/*B. breve*: They have also attracted considerable interest because of their ability to influence gut microbial metabolism and host immune regulation. These anaerobic bacteria contribute to butyrate production and appear to regulate the immunosuppressive indoleamine 2,3-dioxygenase-kynurenine pathway. Compared with many other microorganisms, they possess very low intrinsic pathogenic potential and have been administered safely in both neonatal and immunocompromised populations. Although direct evidence in hematological malignancies remains limited, and most available data originate from preclinical investigations or graft-versus-host disease models, their favorable safety profile and immunomodulatory properties support further evaluation in future hematopoietic stem cell transplantation studies [216].
• *Saccharomyces boulardii*: *S. boulardii* is a probiotic yeast frequently used to prevent antibiotic-associated diarrhea and recurrent *Clostridioides difficile* infection. Despite its widespread use, this organism should not be administered to patients with hematological malignancies who have central venous catheters in place. In 2017, the European Medicines Agency formally revised the product information for *S. boulardii*, concluding that in critically ill or immunocompromised individuals, the potential risks outweigh the anticipated benefits. This decision was based on 61 documented cases of fungemia reported through the EudraVigilance database, nearly half of which occurred in patients carrying central venous catheters. Ten of these cases resulted in death. Current evidence suggests that contamination of catheter hubs during the handling and opening of probiotic capsules is a more likely explanation than direct translocation from the gastrointestinal tract. Because *S. boulardii* products are readily available without a prescription, healthcare providers should actively inform patients about this contraindication, particularly since some individuals may independently use these supplements without reporting their use to the medical team [216].

#### 6.1.2 Risk of probiotic-associated bacteremia and colonization inefficiency

Although probiotic-related bloodstream infections are infrequent, they remain a clinically significant concern in patients with profound immunosuppression. In individuals with hematological malignancies, disruption of the intestinal barrier is common as a consequence of intensive chemotherapy, severe mucositis, and acute graft versus host disease. Under these conditions, probiotic microorganisms may cross the compromised epithelial barrier and gain access to the systemic circulation. This process of microbial translocation is considered the primary mechanism underlying probiotic-associated bacteremia in vulnerable patient populations. Evidence supporting this phenomenon has accumulated over recent years. Kullar et al. (2023) [217] conducted a comprehensive review of reported cases of *Lactobacillus* bacteremia and identified several probiotic strains directly linked to bloodstream infections through molecular characterization. Among the strains most frequently implicated were *Lacticaseibacillus rhamnosus* GG, *Lactiplantibacillus plantarum*, and *Lacticaseibacillus paracasei*. Importantly, advanced molecular typing methods demonstrated that the organisms isolated from blood cultures were genetically indistinguishable from the probiotic strains administered to the affected patients, providing compelling evidence of a causal relationship.

Additional support for this association was provided by the study of Gilliam et al. (2022) [215], which investigated a cluster of bloodstream infections in pediatric hematopoietic cell transplant recipients. Using whole-genome sequencing, the investigators demonstrated a close genetic match between *Lactobacillus* isolates recovered from patients and the concurrently used probiotic products. This report is regarded as one of the strongest demonstrations of probiotic-related bacteremia in a population highly relevant to hematological malignancies and transplantation medicine. The likelihood of developing probiotic-associated bacteremia is not uniform across all patients and appears to increase substantially in the presence of specific clinical risk factors. Severe neutropenia, particularly grade 3 or 4 neutropenia characterized by an absolute neutrophil count below 0.5 × 10^9^/L, markedly reduces host defense mechanisms and increases susceptibility to invasive infections. Likewise, extensive mucosal injury facilitates microbial translocation from the gastrointestinal tract into the bloodstream. The presence of central venous catheters may further increase infection risk by providing a route for microbial dissemination, while the use of broad-spectrum antibiotics can alter the gut microbiota's ecological balance and impair competitive colonization resistance. Together, these factors create conditions that favor both probiotic persistence and opportunistic invasion. Beyond the risk of bacteremia, another important limitation of probiotic therapy in patients with hematological malignancies is its inconsistent colonization efficiency. Successful colonization depends on complex interactions among the administered microorganism, the resident microbiota, host immunity, dietary factors, and concurrent medical treatments. Because chemotherapy, antibiotics, and transplantation procedures profoundly disrupt the intestinal ecosystem, many administered probiotic strains may fail to establish durable colonization. Consequently, clinical benefits observed in one patient population may not be reproducible in another, highlighting the need for individualized approaches and more precise microbiome-guided interventions in future studies.

### 6.2 Fecal FMT: applications and challenges in oncology

The first modern medical application of FMT was reported in 1958, when it successfully treated four patients with pseudomembranous colitis [187,218]. The fastest and most effective method for restoring the gut microbiome is FMT [187]. FMT is the transfer of fecal material from a healthy donor to a recipient to restore the recipient's gut microbial community diversity [190]. In oncology, FMT has been successfully used to eradicate colonization with multidrug-resistant organisms (MDROs) in patients undergoing allo-HCT [190]. In addition, FMT has been used as a therapeutic strategy to treat steroid-resistant acute GvHD and to improve the efficacy of immune checkpoint inhibitors (ICI) within the patient population who have not previously responded to therapy. [183,190,194]. Given the complexity of hematological and oncological diseases and their treatments, these patients may be considered potential candidates for FMT [187].

**Fig. 7:**
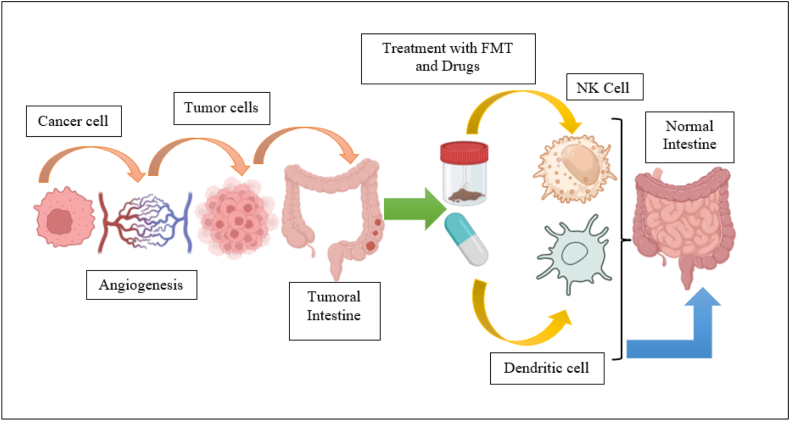
**Schematic representation of hematologic malignancy treatment via gut microbiota modulation.** Beneficial gut microbes, introduced through probiotics or fecal microbiota transplantation (FMT), interact with the host immune system, leading to activation of T cells, natural killer (NK) cells, and dendritic cells (DCs). This immune stimulation enhances the elimination of malignant blood cells, thereby reducing tumor burden. Arrows indicate the direction of interaction and therapeutic effect.

Therefore, FMT has, in recent years, gone beyond the treatment of infectious diseases such as *C. difficile* and has been considered as a promising tool in oncology to control this infection [197]. Findings suggest that targeted microbiome restructuring can alter metabolic and immune pathways, increasing the body's sensitivity to anticancer therapies (Fig. 7) [196].

However, FMT still faces clinical and ethical challenges. Selection of appropriate donors, the risk of transmission of unknown pathogens, standardization of sample processing methods, and determination of optimal dose and frequency are significant concerns [197]. The primary concern regarding FMT in this population is the chance of developing new infections. Concern has increased following reports of Patients with myelodysplastic syndrome who had preventive FMT experienced lethal ESBL *E. coli* septicemia [219]. Such risks are particularly high in patients who are frequently neutropenic, immunosuppressed, and have compromised intestinal barriers. Consequently, FMT in these patients, even for the treatment of *C. difficile* infection (CDI), should be performed only in well-controlled clinical studies. [187]. Additional challenges include the risk of intestinal perforation and gastrointestinal bleeding caused by instrumentation during FMT, especially in patients with intestinal mucositis. The development of capsule-based FMT may help reduce these risks [187]. A recent systematic review of FMT procedures over the past two decades reported the lowest rate of side effects with transendoscopic colonic intubation (6.33%) and the highest with gastroscopy (31.92%), while capsule FMT showed an unexpectedly high rate of 28.97% [220]. Nevertheless, the comparative safety of capsule delivery in hematologic and oncologic patients remains unclear. Therefore, FMT should be performed only within carefully monitored clinical trials in this patient group [187]. The risk of reinfection has also been studied. Overall failure and reinfection rates have been estimated at 14% [221], but repeated FMT significantly reduces these rates in patients treated for CDI [222]. Furthermore, in severely immunocompromised patients, the risk of opportunistic infections remains. To overcome these obstacles, research is now moving towards the use of microbiota consortia (artificial combinations of specific, safe bacteria) and personalized FMT to both maintain immunity and optimize therapeutic effects.

#### 6.2.1 Clinical challenges and potential solutions in FMT for HMs

Despite its biological rationale, the translation of FMT into routine clinical practice for patients with HMs, and particularly for allo-HSCT recipients, requires careful attention to several interrelated challenges: donor selection and microbiome standardization, optimal timing relative to transplant milestones, choice of administration route, and the specific infection risks posed by severe immunosuppression. Addressing each of these systematically is essential before FMT can be adopted beyond carefully monitored investigational settings.

##### 6.2.1.1 Donor selection and microbiome standardization

In the general population, FMT donor screening primarily focuses on excluding enteric pathogens and MDROs. In patients with HMs, however, the threshold for exclusion must be considerably more stringent. A pivotal safety signal emerged in 2019 when DeFilipp et al. reported two immunocompromised patients, one of whom was an allo-HCT recipient, who developed invasive extended-spectrum beta-lactamase (ESBL)-producing *E. coli* bacteremia after receiving FMT from a shared donor whose stool had not been screened for ESBL-producing organisms; one patient died [190]. This report prompted the U.S. Food and Drug Administration (FDA) to issue a safety alert mandating that donor stool be screened at a minimum for ESBL-producing *Enterobacteriaceae*, vancomycin-resistant *Enterococcus* (VRE), carbapenem-resistant *Enterobacteriaceae* (CRE), and methicillin-resistant *S. aureus* (MRSA) before use in any clinical investigation.

In the hematological malignancy setting, contemporary consensus panels additionally recommend serological and molecular testing for hepatitis A, B, C, and E viruses; HIV; cytomegalovirus (CMV); Epstein-Barr virus (EBV); HTLV-1/2; *Treponema pallidum*; and *Strongyloides stercoralis*, the latter being particularly important given the risk of hyperinfection syndrome under prolonged immunosuppression. SARS-CoV-2 PCR stool screening has been incorporated into more recent protocols. Donors should also be deferred from donating if they have used antibiotics, immunosuppressants, or chemotherapy within the preceding 3 months, and a comprehensive travel and dietary history should be obtained.

Beyond pathogen exclusion, an important and unresolved question concerns the optimal microbiome composition of the donor preparation. Observational data from allo-HSCT cohorts suggest that a higher donor abundance of SCFA-producing taxa, particularly Blautia spp., *F. prausnitzii*, and *Roseburia intestinalis*, is associated with a lower risk of GVHD and improved survival. However, no validated, prospectively tested criteria currently guide donor selection based solely on microbiome composition; this remains an important priority for future trial design.

### 6.3 Personalized microbiome-based therapies: prospects for tailored interventions

It is important to note that individual differences in microbiota composition can be attributed to a variety of factors, including environmental factors, drug use, and food [29].

Therefore, the future of microbiome modulation in cancer therapy lies in the development of personalized interventions [101]. This involves the use of defined bacterial consortia, consisting of specific beneficial strains, rather than the undefined community delivered in FMT [190]. Such “next-generation” probiotics could be targeted to address deficiencies in a patient's specific microbiome and immune function, or to increase drug metabolism [101]. Finally, a deeper understanding of host-microbe interactions enables the development of microbiome-based personalized therapies to improve treatment efficacy and decrease mortality for patients with HMs [10]. Progress in pharmacomicrobiomics is enabling microbiome-based, personalized interventions tailored to a patient's microbiota, treatment regimen, and risk profile [40,223]. The composition of the baseline gut microbiome could predict infection risk during chemotherapy and guide targeted probiotic or prebiotic interventions [37].

Dysbiosis Patterns in AML are associated with treatment toxicity, showing that interventions to return barrier integrity or modulate metabolite production can be personalized [223]. Donor- or autologous-derived FMT, based on proper selection, is possible and safe [40]. Challenges include individual diversity, antibiotic effect, diet, immune status, and logistical barriers. Trials are needed to define optimal timing, dosage, formulation, and long-term stability [40,223].

In summary, microbiome-based personalized interventions in leukemia are promising for predicting risk, regulating the microbiome, optimizing care, reducing toxicity, and improving treatment response, although further validation is needed. The development of “omics” technologies, including metagenomics, metabolomics, and transcriptomics, has enabled a more precise understanding of the complex interactions between the microbiome and cancer treatment. Based on these data, personalized therapeutic approaches are being developed to deliver interventions tailored to each patient's characteristics [185]. In this field, patients' microbial patterns can be used to predict response to chemotherapy or immunotherapy. For example, high *Firmicutes* diversity and presence of *Faecalibacterium* before treatment have been associated with a higher possibility of response to anti-PD-1 Barragtherapy [224]. Ongoing interdisciplinary research is essential to uncover the full clinical potential of targeted microbiota interventions and to pave the way for more effective, personalized cancer treatments [29].

On the other hand, machine learning models are being developed that, using microbial and clinical data, can predict the most appropriate intervention (probiotics, FMT, or diet) for each patient [205]. In parallel with these advances, new research is moving toward the use of engineered bacteria to transmit drugs or modulate immune responses. These approaches could transform the microbiome into a living platform for the targeted therapies delivery [202]. Although these technologies are still in their early stages, they could represent a milestone in cancer treatments in the near future. Ultimately, microbiome-based personalized therapies will only be successful if they are supported by standardized sampling, data analysis, and clinical trials. The future of oncology is clearly moving towards integrating microbiome data with patient genomics and metabolomics, an approach that will elevate the concept of Precision Oncology to a new level by combining interdisciplinary research and machine learning.

### 6.4 Shared versus disease-specific microbiota modulation strategies: A synthesis for clinical translation

The evidence reviewed across Sections 1.1, 1.3 reveals both convergent and divergent microbiota-disease relationships among the major hematological malignancy subtypes. Before considering targeted interventions, it is instructive to distinguish which therapeutic strategies address universal dysbiosis features, applicable across the spectrum of HMs, from those that are mechanistically tied to the biology of a specific tumor type. This distinction is clinically relevant because it determines whether interventions can be protocolized broadly or require disease-specific adaptation.

#### 6.4.1 Shared microbiota features and common gut-modulating strategies

A consistent pattern of gut dysbiosis is observable across all hematological malignancy subtypes reviewed (see Table 1). Three convergent features emerge with particular regularity:

• Depletion of SCFA-producing taxa: Reduced levels of *F. prausnitzii* and related *Firmicutes* are reported in AML, ALL, HL, NHL, and MM, leading to diminished butyrate production. Butyrate exerts epigenetic anti-tumor effects via HDAC inhibition, reinforces the intestinal barrier, and promotes Treg differentiation. Its depletion across subtypes represents a shared vulnerability amenable to common intervention.
• Elevated systemic pro-inflammatory cytokines (IL-6, TNF-alpha): Elevation of these cytokines, documented in AML, ALL, CML, CLL, HL, and MM, reflects a shared dysbiosis-driven inflammatory state mediated by LPS-TLR4-NF-kB signaling and reduced anti-inflammatory metabolite production. Strategies targeting this axis have relevance across disease types.
• Disruption of mucosal barrier integrity: Chemotherapy- and disease-related barrier disruption, coupled with endotoxemia, is a universal complication that amplifies systemic inflammation and infection risk and is independent of specific tumor genetics.

#### 6.4.2 Tumor-type-specific microbiota modulation strategies

Beyond these shared approaches, several mechanistically grounded, disease-specific microbiota-host interactions identified in this review suggest that certain modulation strategies should be tailored to the tumor biology of individual subtypes:

##### 6.4.2.1 CML and the BCR-ABL1–microbiota interplay

CML represents the clearest example of a tumor-specific driver directly intersecting with microbiota biology. The BCR-ABL1 fusion gene drives constitutive tyrosine kinase activity, and its first-line treatment with NAMPT inhibitors (such as APO866) is directly undermined by gut bacteria capable of converting nicotinamide (NAM) to nicotinic acid (NA), thereby fuelling an alternative NAD biosynthesis pathway that rescues leukemia cells from NAD depletion [47]. This interaction is not relevant to other hematological malignancy subtypes and has direct translational implications: targeted modulation of the microbiota, including antibiotic-mediated suppression of NAM-to-NA-converting bacteria or dietary NAM restriction, may be necessary to preserve NAMPT inhibitor efficacy in CML patients. Additionally, TKI-induced gastrointestinal side effects (diarrhea, altered intestinal motility) in CML patients create bidirectional dysbiosis that warrants disease-specific monitoring and probiotic support protocols. An ongoing clinical trial (NCT06724536) is prospectively evaluating microbiome signatures associated with deep molecular response to TKIs in CML.

##### 6.4.2.2 MM: the *P. heparinolytica*-Th17-BM axis

In MM, a disease-specific microbiota-immune circuit has been mechanistically established in preclinical models: *P. heparinolytica* promotes intestinal Th17 cell differentiation, and these gut-primed Th17 cells migrate to the BM, where IL-17 production activates eosinophils and triggers IL-6 release, sustaining plasma cell proliferation and accelerating progression from smoldering to symptomatic MM [63,64]. Conversely, *Prevotella melaninogenica* restrains MM progression by limiting Th17 expansion. This axis, absent in the other subtypes reviewed, suggests disease-specific therapeutic opportunities: targeted suppression of *P. heparinolytica*, dietary strategies to reduce its substrate availability, or IL-17/IL-17R blockade as adjuncts to standard myeloma therapy. Furthermore, the increase in nitrogen-recycling bacteria (e.g., *Klebsiella pneumoniae*) that fuels glutamine synthesis for plasma cell energetics is a MM-specific metabolic vulnerability that may guide future nutritional or prebiotic interventions [63].

##### 6.4.2.3 CLL: TLR-BCR crosstalk and the effect of BCR inhibitors on the microbiome

In CLL, TLR ligands derived from gut microbiota can co-stimulate BCR signaling in malignant B cells, a crosstalk that is mechanistically absent in myeloid leukemias. Ibrutinib and other BTK inhibitors, by blocking BCR-downstream NF-kB signaling, reduce homing signals for CLL cells and alter the tumor microenvironment. Concurrently, BCR inhibitor therapy has been shown to increase Bacteroidia abundance in CLL patients [41], suggesting that targeted therapy itself reshapes the microbiome. This bidirectional relationship, where both the tumor's signaling pathway and its treatment modify microbiota composition, is distinct to CLL and implies that microbiome monitoring and support during BTK inhibitor therapy should be regarded as a disease-specific rather than a generic oncological recommendation.

##### 6.4.2.4 Acute lymphoblastic leukemia (ALL, especially pediatric): pre-treatment microbiome as a predictor of chemotherapy tolerance

In ALL, particularly in children, the pre-treatment microbiome composition has specific predictive value for chemotherapy tolerance and infectious outcomes, a value that has not been as consistently demonstrated in adult myeloid malignancies. The predominance of *Proteobacteria* and *Enterococcus* before treatment initiation specifically predicts infection risk and diarrheal complications during induction. This creates an opportunity for ALL-specific pre-treatment microbiome profiling and targeted probiotic priming strategies (e.g., SCFA-producing organism supplementation before induction) that are not yet supported for other subtypes. The persistence of microbiome alterations beyond chemotherapy completion, as described in pediatric ALL cohorts [36,37], also necessitates ALL-specific long-term survivorship microbiome monitoring protocols that are not currently incorporated into adult hematology practice.

## 7 Methodological considerations in microbiota research

### 7.1 Study designs and approaches: Strengths and limitations of current research methodologies

Biological processes that govern health and disease are inherently dynamic and are best understood when analyzed in a temporal context [225]. Identifying specific gut microbiota members that causally contribute to human diseases has proven challenging, as interindividual variability in microbiota composition can mask differences between healthy and diseased individuals, particularly in cross-sectional study designs [226]. Cross-sectional studies are often used to investigate the structure of the microbiota at a specific point in time. These investigations represent a rapid and effective manner to examine the correlation between microbial profiles and health outcomes. However, because these studies cannot demonstrate a temporal relationship between changes in the microbiota and the onset of disease, they are limited in their ability to infer causality [227].

On the other hand, because longitudinal studies follow patients over time, they allow researchers to observe how the microbiota evolves dynamically and how this may relate to the course of a disease or the effectiveness of treatment. Understanding how nutritional, drug, or lifestyle interventions affect microbial communities is much easier with such designs. However, longitudinal studies can be biased since they are time-consuming, resource-intensive, and prone to participant drop; therefore, such designs need to. Follow up on long-term and accurate control of variables [225].

RCTs, or randomized controlled trials, are the gold standard for evaluating how interventions affect the microbiome. RCTs improve the reliability of results by reducing bias through random assignment to intervention and control groups. However, diagnosing consistent intervention effects may be complicated by significant variability in microbiome composition among individuals [228].

Interventional studies are typically conducted on a smaller scale than national studies because they allow for greater experimental control through randomization and the use of patient groups. Such studies often follow a prospective, placebo-controlled design, which enables systematic monitoring of participants over time. By collecting microbiome samples at multiple stages during the study, researchers can investigate how the microbiome shifts following interventions. This design provides a robust framework for establishing causal links between alterations in the microbiota and clinical outcomes, supported by advanced analytical approaches, including metagenomics and other omics-based technologies [229].

To investigate mechanistic links, microbial populations can be carefully manipulated in animal models, such as GF and gnotobiotic models. They are highly specialized in preclinical research to understand microorganisms that affect human physiology. However, the findings may not be as widely applicable due to changes in human and animal physiology and microbiomes [230].

Large-scale associations between microbial characteristics and host phenotypes can now be found using microbiome-wide association studies (MWAS). The high complexity and dispersion of microbiome data increase the possibility of false-positive results, even though MWAS can identify possible biomarkers. To guarantee reliable results, accurate statistical corrections are essential, including validation cohorts and adjustments for multiple testing [231].

The study of the microbiome presents significant statistical and analytical difficulties. The heterogeneous, dispersed nature of microbiome data violates the assumptions of many standard statistical techniques. To take these traits into account and increase interpretability, complex methods such as compositional data analysis, Bayesian hierarchical models, and machine learning techniques are being increasingly used [232]. To decrease these problems, it is recommended to use a multi-center design, random sampling, and metadata-accurate documentation [233].

One approach in therapeutic engineering is to change mechanical pathways to alter treatment outcomes. Choudhury and colleagues developed a genetically engineered non-pathogenic *E. coli* strain designed to indirectly promote tumor regression by boosting the host immune system. The bacteria were modified to engineer a synchronous lysis circuit in their genome, enabling the release of a nanobody-encoded CD47 antagonist (CD47nb), which is the anti-phagocytic receptor on cancer cells. This strategy stimulates T-cell activation, leading to tumor shrinkage and potentially preventing metastasis. Overall, the simultaneous lysis rate circuit has an increased impact on treatment outcomes in patients with cancer, although this approach remains to be explored [234].

Combination therapies remain effective in cancer prevention, but future drug trials must account for interactions among genetic (genomic), environmental (exposome), and microbiome factors. This is particularly challenging because numerous xenobiotics affect not only the composition but also the functional potential of the gut microbiota [150].

Most interventional studies conducted so far have used animal models, which have provided important insights into microbiome-drug interactions. However, differences in microbial composition and immune responses between mice and humans limit the direct translation of these findings to clinical practice [235,236]. Therefore, large-scale human studies are necessary to confirm these results in clinical settings.

FMT has emerged as a promising therapeutic approach with multiple potential applications. Nevertheless, concerns remain regarding its use in patients with hematological and oncological diseases, particularly due to “donor effects,” which depend heavily on the donor-specific microbiota composition. Such variability complicates standardization and may lead to unintended microchanges in all populations, with adverse effects. Hence, FMT in these populations should be performed only in well-controlled clinical trials [29].

The Bacillus Calmette-Guerin (BCG) vaccine has been shown to stimulate hematopoietic stem and progenitor cell (HSPC) differentiation, enhancing myelopoiesis via TLR-dependent mechanisms [120,121,237,238]. However, prolonged TLR activation from chronic infection or dysbiosis dominated by pathogenic bacteria can negatively affect HSPC function [120,121,237]. Furthermore, vaccine efficacy has been linked to the composition of the gut microbiota [239], suggesting that integrating microbiota modulation with cancer vaccination may offer a novel strategy to enhance therapeutic outcomes. Personalized cancer immunotherapy may become increasingly feasible as research progresses.

Beyond the gastrointestinal tract, the oral microbial ecosystem, skin, and benign tumor tissue can also shape immune responses, broadening opportunities for personalized treatment strategies. [240,241]. The interaction between the gut microbiota and immunotherapy is complex: the gut microbiota can influence immunity by modulating inflammatory processes through metabolite-mediated mechanisms. These effects are bidirectional, as certain microbes can enhance immune responses while others may suppress them. Furthermore, changes in the tumor microenvironment (TME) complicate interactions [242].

Future research should aim to identify essential bacterial strains and their metabolic byproducts for the application of microbiota-targeted strategies that improve the effectiveness of immunotherapy. However, identifying consistent and clinically useful microbial markers is challenging due to individual variability, environmental influences, variations in clinical trial design, and methodological differences [29].

### 7.2 Challenges in microbial profiling: variability in sequencing techniques and data interpretation

Mistakes in sample handling, variations in sample size, choice of DNA extraction methods, library preparation, and bioinformatics algorithms can lead to inconsistent results, and one of these factors is a main contributor to differences across numerous studies [233,243]. Microbial profiling through metagenomic gene analysis has shown significant issues, including unreliability of classification at the species level, and biases induced by sequencing platforms and the selection of diverse data processing software.” [233].

Similarly, the use of analytical pipelines to process data may lead to differences in microbiota composition, as not all available pipelines include the same steps [233]. However, more complex bioinformatics pipelines represent comprehensive insights into microbial diversity and functional genes [244]. Study results are significantly influenced by the selection of sequencing method, including whole-genome sequencing, shotgun metagenomics, and 16S rRNA gene amplicon sequencing. Each technique has its own advantages and disadvantages [244]. Results may be affected by methodological differences between laboratories. The need for standardized methods in microbiome research was emphasized by Forry et al. (2024), who showed that laboratories using numerous techniques produced different classification profiles [245].

The absence of standardized research protocols contributes to variability in microbiome data, and the field faces several important challenges:

First, technical differences in microbiome sequencing, including variations in the 16S rRNA gene regions analyzed, sample preparation and DNA extraction methods, and sequencing platforms, can create notable discrepancies between studies [246,247]. Among these, 16S rRNA gene sequencing remains the most commonly used approach, as it enables bacterial identification at the genus level at a reasonable cost [244,248]. However, this approach has certain drawbacks, such as limited sensitivity for distinguishing between bacterial species and reliance on the selected target region (V3–V4) [249]. In contrast, shotgun metagenomic sequencing, although more costly and technically demanding, provides a more comprehensive and detailed understanding of microbial diversity and functional gene profiles [245]. The shotgun metagenomic approach represents more accurate functional information with more complete reads, but is more expensive and complex [250]. Recently, long-read technologies such as PacBio and Oxford Nanopore have increased the accuracy of identification [251].

Second, differences in study design, such as patient recruitment criteria, sample processing, and data analysis methods, can reduce reproducibility and make comparisons across trials more difficult [252]. Host genetics also strongly influences microbiota composition and immune responses, but these factors are often overlooked in current research designs [253]. In addition, the lack of standardized protocols for microbiome analysis limits comparability between studies, and safety considerations add further complexity in clinical contexts [29].

The microbiome dataset is compositional, in that an apparent rise in bacterial species does not necessarily indicate an absolute increase. This characteristic complicates data interpretation and requires careful analytical approaches. Therefore, the use of models with traditional data, along with proper appropriateization techniques, is crucial for reliable analysis. Small sample sizes in many studies limit statistical power and reduce the ability to generalize findings. Overall, achieving standardization remains challenging because gut microbiota composition varies widely between individuals due to factors such as diet, medication use, and environmental exposures [29].

### 7.3 Confounding factors: impact of diet, lifestyle, and medications on study outcomes

For many prevalent and complex human diseases, the inclusion of well-matched control groups helps minimize confounding variables, clarify observed differences in microbiota, and reduce the risk of spurious associations [226].

#### 7.3.1 Dietary

Among environmental factors, diet is a key driver of the gut microbiota. Studies have demonstrated that distinct dietary patterns, such as high-fiber or high-fat diets, can lead to characteristic microbial profiles [254]. Following Western or traditional dietary patterns can have widespread consequences for microbial balance and overall health [255]. Dietary habits also play an important role in shaping the gut microbiota. For instance, individuals consuming a Western diet typically show a predominance of *Bacteroides* species.

Furthermore, diets high in animal fats but low in fiber and plant-based protein have been associated with an increased risk of cancer, primarily due to the accumulation of bile acids and reduced production of SCFAs by gut bacteria [200,254].

While traditional plant-based diets are associated with higher concentrations of degrading fiber-degrading bacteria [254], dietary changes may temporarily alter the composition. Short-term dietary treatments often induce rapid and transient increases in microbial diversity, and the microbiota returns to baseline after the diet is discontinued [256]. This change underscores the importance of considering long-term dietary trends in microbiome research.

#### 7.3.2 Lifestyle

Lifestyle factors such as stress levels, sleep and exercise habits, and Alcohol consumption and contact with pets may have been altered. While sedentary behavior and long-term stress can cause dysbiosis, defined by a decrease in microbial diversity and an overgrowth of pro-inflammatory bacteria, Regular exercise has been linked to increased microbial diversity and the presence of beneficial species [226,257,258]. Lifestyle factors, such as the frequency of alcohol consumption and bowel movement patterns, have also been identified as significant and sometimes unexpected contributors to gut microbiota variation. These differences can vary between healthy and diseased individuals, potentially confounding study results [226]. These lifestyle-related changes can complicate the interpretation of microbiome findings when the above-mentioned are not adequately controlled in study designs.

#### 7.3.3 Effect drugs

In addition, the composition of the gut microbiota can be substantially influenced by medications, particularly antibiotics, proton pump inhibitors (PPIs), and nonsteroidal anti-inflammatory drugs (NSAIDs), which may alter microbial diversity and metabolic activity. Antibiotics disrupt microbial ecosystems by decreasing diversity and encouraging the emergence of opportunistic infections [259].

Certain medications can also alter microbial populations. NSAIDs and PPIs have both been shown to induce microbial shifts that may negatively affect health. PPIs, in particular, are frequently prescribed to hematology and oncology patients experiencing marked thrombocytopenia and mucositis after chemoradiotherapy, to minimize the probability of hemorrhage in the gastrointestinal tract [187].

Both drugs and underlying disorders can simultaneously affect microbiota composition, making it difficult to separate their effects [260]. Therefore, controlling these variables is essential in study design. Researchers recommend that accurate information on participants' diet, drug consumption, and daily habits be recorded at the time of sample collection. In data analysis, the confounding effect of these variables can also be decreased when they are used as cofactors. It is also recommended to perform a sensitivity analysis to evaluate the stability of the results (Table 4) [244].

**Table 4:** **Overview of Pathogenesis, Gut Microbiota Alterations, and Therapeutic Strategies in HMs.** N/A, not applicable.

Feature	Healthy State	(AML)	(ALL)	(CML)	(CLL)	(HL)	(NHL)	(MM)	Reference
Primary Etiology	N/A	Malignancy of defective HSCs	Malignant lymphoid progenitor proliferation	Philadelphia chromosome & BCR-ABL1 fusion	Slow-progressing leukemia linked to B-cell microenvironment	Abnormal Reed-Sternberg (HRS) cells	Heterogeneous B/T-cell malignancies	Plasma cell malignancy	[25,33,40,43,51,52,59]
Hematopoietic Outcome	Normal production of immune cells through HSC differentiation	Rapid multiplication of abnormal myeloid cells in the BM	Lymphoid progenitor proliferation in BM, blood, and extramedullary areas	BCR-ABL1 kinase-driven proliferation	Slow progression	Loss of typical B-cell phenotype	Malignant proliferation of B or T lymphocytes	Plasma cell accumulation & marrow infiltration	[7,33,40,43,52,59,60]
Gut Microbiota Diversity	Generally diverse and functionally constant	Pronounced depletion of bacterial diversity	Significant decline in bacterial diversity	Reduction of the Tenericutes phylum	Immune-linked signatures; therapy-driven *Bacteroidia*	Decreased rare gut bacteria/Low alpha-diversity	Reduced alpha-diversity	Gut flora alterations linked to relapse	[5,10,14,40,48,53]
Dysbiosis	N/A	Reduction of the *Eubacterium* Genus, especially *Eubacterium eligens*	*Proteobacteria/Enterococcus* dominance, *B. clarus* enrichment, SCFA depletion	Increased *Actinobacteria, Acidobacteria, Chloroflex, E. coli;* reduced *Tenericutes*	Immune-linked signatures; BCRi-induced *Bacteroidia* elevation	*F. prausnitzii* depletion	Increased *Escherichia* and *Shigella,* and *Lachnospira,* reduced *F. prausnitzii*	relapse risk by Eubacterium	[7,30,31,[36], [37], [38],^40^,^41^,[47], [48], [49],^53^]
Intestinal Barrier Integrity	Maintained mucosal barrier	Barrier deficiency linked to infection & inflammation	Chemotherapy-induced mucositis	TKLs-induced barrier defect	Increased intestinal permeability	Chemotherapy-induced mucosal injury	Chemotherapy-induced barrier disruption	Microbiota-associated mucosal injury	[2,7,15,30,31,35,98,156]
Systemic Inflammation	Immune homeostasis limits inflammation.	Elevated serum IL-6 & TNF-α	Elevated serum IL-6 & TNF-α	Elevated IL-6 & TGF-α	Elevated TNF-α & IL-6	High IL-6/TNF-α & B symptoms	Elevated serum IL-6 & IL-10	NF-κB & cytokine-driven microenvironment	[2,7,[30], [31], [32],^35^,^44^,^54^,^55^,^57^,^59^,^91^]
Pro-inflammatory Cytokines	Immune homeostasis limits inflammation	Elevated serum IL-6 & TNF-α	Elevated serum IL-6 & TNF-α	Elevated IL-6 & TNF-α levels	Elevated TNF-α & IL-6	Elevated IL-6 & TNF-α	Elevated IL-6 & IL-10	Elevated BM IL-6 & VEGF	[2,7,[30], [31], [32],^54^,^57^,^59^,^91^]
Malignant Cells in Blood/BM	N/A	Abnormal myeloid cells	Lymphoid progenitor cells	Cells with BCR-ABL1 fusion	Leukemic B-cells	Reed-Sternberg (HRS) cells	B or T lymphocytes	Abnormal plasma cells	[29,33,43,51,52,55,59]
Treatment Considerations	N/A	Chemotherapy & antibiotics	Chemotherapy	TKIs & Allo-HSCT	BCRi	Chemo-radiotherapy	Subtype-specific Chemo/Immunotherapy	PIs, IMiDs & Transplant	[7,31,36,41,42,45,46,51,57,58]
Microbiota-Targeted Therapies	Pro/Prebiotics	FMT	Probiotics	Antibiotic-induced microbiota depletion, e.g, *E.coli*) (to enhance APO866 efficacy)	Targeted microbiota modulation	FMT & Probiotics	FMT & Probiotics	FMT	[[15], [16], [17],^40^,^47^,^162^,^196^,^202^]

## 8 Future directions for research and therapeutic innovation

Selecting an appropriate study design, using standard technologies, and controlling for confounding variables are vital to increasing the accuracy and reproducibility of studies.

Applying microbiome analysis to cancer and hematology could revolutionize our knowledge and approach to treating these diseases, revealing the complex relationships between bacteria and cancer cells, creating more accurate diagnostic tools and personalized treatments that will help promote the harmonious symbiosis of the human body and its microbial environment, and thereby promoting long-term health and wellness. For example, strategies such as probiotics and prebiotics have been proposed to restore microbial balance and reduce treatment-related side effects, although their safety in severely immunocompromised patients remains a challenge and needs careful evaluation. FMT, as the most efficient approach for microbiome restoration, shows significant therapeutic potential for steroid-refractory GvHD and for improving immunotherapy responses. However, significant safety challenges, especially the risk of pathogen transmission in neutropenic patients, limit its use and necessitate well-controlled clinical trials. Furthermore, examining the microbiota's role in regulating innate and adaptive immune responses and assessing its impact on the efficacy of targeted treatments and immunotherapeutic strategies is a new research direction. There is mounting evidence that the gut microbiota's composition can affect how patients respond to immune checkpoint inhibitors and may predict treatment susceptibility or resistance. In this regard, the discovery of microbiome-based biomarkers holds great promise for improving patient classification, facilitating more accurate treatment selection, and reducing unnecessary costs and side effects.

Simultaneously, the complex interactions among the microbiota, microbial-derived compounds, and host cellular signaling pathways represent a significant breakthrough in developing innovative treatment approaches. SCFAs, tryptophan-derived metabolites, and secondary bile acids are essential for controlling inflammatory responses, directing immune cell development, and preserving mucosal barrier integrity.

In patients with hematologic malignancies, whose immune balance is extremely delicate, targeting these metabolic pathways may lead to more targeted therapeutic approaches with fewer adverse effects than traditional systemic medications.

SCFAs, such as acetate, propionate, and butyrate, are among these metabolites that have immunomodulatory effects through epigenetic mechanisms, HDAC inhibition, and promotion of Treg-cell differentiation. These mechanisms help to control inflammation and maintain intestinal epithelial integrity. Similarly, by activating the aryl hydrocarbon receptor (AhR) pathway, metabolites produced by tryptophan metabolism regulate mucosal immunity and maintain equilibrium between pro-inflammatory and anti-inflammatory responses, thereby limiting excessive immunological activation. Secondary bile acids also affect T-cell activity, macrophage polarization, and cytokine secretion profiles by interacting with nuclear and G protein-coupled receptors.

Together, these metabolic and signaling pathways are essential for maintaining immunological homeostasis and preventing chronic inflammation, and their dysregulation may contribute to the emergence of autoimmune, inflammatory, and cancerous disorders. Therefore, rational targeting of these pathways opens new possibilities for creating more targeted and individualized therapeutic interventions, whether through direct targeting of host receptors, altering microbial metabolite synthesis, or altering microbiota composition. These methods have a lower risk of side effects than traditional systemic treatments, and they are especially promising for patients with hematologic malignancies, whose immune balance is severely disrupted by both the disease and intensive treatment regimens.

Furthermore, advancements in next-generation sequencing technologies, functional metagenomics, metatranscriptomics, and metaproteomics have enabled a more thorough examination of microbiota dynamics over time and in response to therapeutic interventions. Predicting disease progression, therapeutic response, and even the occurrence of treatment-related adverse events may be made easier by integrating these multilayered datasets with clinical and genetic patient data using artificial intelligence and deep learning models. This is an important step toward predictive and personalized medicine.

Hence, the international Compilation of instructions and shared data banks, personalized treatments, the use of “defined bacterial consortia” (next-generation probiotics), and the use of “Omics” technologies and machine learning models to design accurate interventions based on each patient's microbial profile could be important steps towards advancing the quality of future research. Microbiota research has represented new perspectives in personalized medicine and disease prevention, and future horizons focus on transforming these findings into therapeutic interventions. Of course, achieving the sustainable and effective development of microbiota-based therapies requires a simultaneous commitment to both scientific rigor and ethical responsibility.

A critical limitation of the current evidence base is the predominantly associative nature of human microbiome and hematological malignancy studies. Cross-sectional and retrospective designs dominate the literature, and the directionality of the observed dysbiosis, whether it precedes or follows disease onset and treatment, remains largely unresolved. Randomized controlled trials with pre-registered microbiome endpoints, standardized profiling protocols, and clinically meaningful primary outcomes are urgently needed before microbiome-based interventions can be incorporated into routine hematological malignancy care.

## 9 Conclusion

Current narrative review demonstrates that the microbiota plays a vital and multifaceted role in the onset, course, and management of HMs. The gut microbiota plays a crucial role in the development and regulation of the host's innate and adaptive immune responses, and, in particular, influences inflammation, immunological regulation, and cellular metabolism, acting as a critical mediator between the immune system and the external environment and influencing hematopoiesis (production of blood cells). Microbial imbalance, or dysbiosis, is another key contributor to carcinogenesis, promoting chronic inflammation and disrupting microbial metabolism, for example, by reducing the levels of protective SCFAs.

Furthermore, microbiota composition plays a decisive role in determining treatment responses. It also has the potential to modulate both the effectiveness and side effects of chemotherapy and modulate a patient's responsiveness to immunotherapy. Evidence from recent studies suggests that patients with a stable and diverse microbiome often experience faster recovery and better tolerance to cancer therapies, highlighting the potential of microbiome-based approaches in personalized medicine.

In allo-HSCT, maintaining the integrity of the gut microbiome is essential for favorable patient outcomes. A reduction in microbial diversity following transplantation has been directly linked to a higher risk of GvHD and increased mortality.

## Ethical approval

This article does not contain any studies with human participants or animals performed by any of the authors.

## Data availability statement

No new data were generated.

## Use of AI statement

Artificial intelligence assistance (OpenAI ChatGPT, GPT-4) and Grammarly were used exclusively for language editing, translation, grammar refinement, and formatting. The authors reviewed and verified all content to ensure accuracy and integrity of the final manuscript. Furthermore, we used Biorender (Free version, online) to draw the Figures.

## Funding

This research did not receive any specific grant from funding agencies in the public, commercial, or not-for-profit sectors.

## CRediT authorship contribution statement

**Atefeh Valaei:** Conceptualization, Data curation, Supervision, Writing – original draft, Writing – review & editing. **Neda Zahmatkesh:** Data curation, Writing – original draft, Writing – review & editing. **Romina Aghaei:** Data curation, Writing – original draft, Writing – review & editing. **Mahtab Maleki:** Data curation, Writing – original draft, Writing – review & editing. **Maryam Meskini:** Conceptualization, Investigation, Project administration, Supervision, Validation, Writing – original draft, Writing – review & editing. **Seyed Davar Siadat:** Conceptualization, Investigation, Project administration, Supervision, Validation, Writing – original draft, Writing – review & editing.

## Declaration of competing interest

The authors declare that they have no known competing financial interests or personal relationships that could have appeared to influence the work reported in this paper.
